# Assessment of suitable habitat of mangrove species for prioritizing restoration in coastal ecosystem of Sundarban Biosphere Reserve, India

**DOI:** 10.1038/s41598-022-24953-5

**Published:** 2022-12-05

**Authors:** Mehebub Sahana, Gopala Areendran, Haroon Sajjad

**Affiliations:** 1 School of Environment, Education and Development, University of Manchester, Oxford Road, Manchester, M13 9PL UK; 2 IGCMC, WWF-India, New Delhi, India; 3 Department of Geography, Faculty of Natural Sciences, Jamia Millia Islamia, New Delhi, India

**Keywords:** Ecology, Ecology, Environmental sciences

## Abstract

Mangrove forests being the abode of diverse fauna and flora are vital for healthy coastal ecosystems. These forests act as a carbon sequester and protection shield against floods, storms, and cyclones. The mangroves of the Sundarban Biosphere Reserve (SBR), being one of the most dynamic and productive ecosystems in the world are in constant degradation. Hence, habitat suitability assessment of mangrove species is of paramount significance for its restoration and ecological benefits. The study aims to assess and prioritize restoration targets for 18 true mangrove species using 10 machine-learning algorithm-based habitat suitability models in the SBR. We identified the degraded mangrove areas between 1975 and 2020 by using Landsat images and field verification. The reserve was divided into 5609 grids using 1 km gird size for understanding the nature of mangrove degradation and collection of species occurrence data. A total of 36 parameters covering physical, environmental, soil, water, bio-climatic and disturbance aspects were chosen for habitat suitability assessment. Niche overlay function and grid-based habitat suitability classes were used to identify the species-based restoration prioritize grids. Habitat suitability analysis revealed that nearly half of the grids are highly suitable for mangrove habitat in the Reserve. Restoration within highly suitable mangrove grids could be achieved in the areas covered with less than 75 percent mangroves and lesser anthropogenic disturbance. The study calls for devising effective management strategies for monitoring and conserving the degraded mangrove cover. Monitoring and effective management strategies can help in maintaining and conserving the degraded mangrove cover. The model proves to be useful for assessing site suitability for restoring mangroves. The other geographical regions interested in assessing habitat suitability and prioritizing the restoration of mangroves may find the methodology adopted in this study effective.

## Introduction

Mangrove forests play a significant role in maintaining the health of the coastal ecosystems globally and support life for birds, flora, and fauna^1–4^. Healthy mangrove forests protect the coastal ecosystem against natural hazards and are a great source of carbon sequestration than any other type of forest^5,6^. The mangrove forests provide significant ecological, social, and economic services to the coastal communities^7,8^. Mangrove forests are one of the most productive ecosystems in the world, being the abode of diverse fauna and flora^9–11^. In spite of providing immense benefits, these ecosystems are under constant degradation due to anthropogenic disturbance and climate change^12,13^. Nearly half of the mangrove biomes have been despaired since 1950 due to huge habitat alteration and inadequate protection^14^. It is anticipated that if this rate of loss is continued then the mangrove biome will be vanished from the earth by the next 100 years^2^. A recent study by Polidoro et al.^15^ on mangrove species over the world reported that more than 16% of species are in the state of becoming extinct and another 10% are under threat of degradation. The mangroves experience higher losses than the average losses of the tropical and sub-tropical^12,16^.

India has a total area of 4975 km^2^ under mangroves which constitutes 0.14% of the country’s total geographical area^17^. The Sundarbans has the largest halophytic mangrove forest in the world and spread over Ganga- Brahmaputra and Meghna delta. The Indian Sundarban Biosphere Reserve (SBR) is a significant ecological region due to its luxuriant mangrove forest and high biodiversity^18^. However, the area under mangrove forests has sharply decreased during the last few decades^19^. Sea level rise, sudden disaster events, over-harvesting, aquaculture expansion, shrimp and salt farming, regular oil spills, and lack of sustainable adaptative strategies may be attributed to the decline of forests^20–22^. Nearly 76% area under *Heritiera fomes* species has declined *during* 1959–2005^23,24^. The other dominant mangrove species in Sundarbans namely *Ceriops decandra, Excoecaria agallocha* and *Xylocarpus mekongensis* also reported having a higher rate of decline^25,26^.

Limited information on the habitat distribution of mangrove species has restricted successful mangrove conservation^27–29^. Advancements in geospatial technology have helped in providing spatial, comprehensive, reliable, and up-to-date information on forest dynamics for effective management^30–32^. Species distribution models (SDM) supported by geoinformatics and machine learning-based spatial analysis have been widely used for mapping, conserving, and management of endangered, threatened, and vulnerable species^33–35^. SDM is a very powerful tool for identifying suitable habitats for the present scenarios and future predictions creating a relation between the known occurrence data and environmental and climatic variables^36–38^.

Different mangrove restoration programmes were promulgated over the last 50 years^39^. During 1980, silviculture and reforestation were programmes were suggested for the mangrove restoration^40^. Later, ecological mangrove restoration (EMR) and community-based restoration approaches were introduced in 2000^41^. Presently, ecological engineering and ecosystem design are one of the most popular mangrove restoration approaches^42^. Romañach et al.^43^ recommended that integrated community-based balances conservation goals are very necessary for conservation and restoration of the mangrove. Sulochanan et al.^44^ used water and sediment quality parameters based pragmatic approach to restore the mangrove ecosystems in Dakshina Kannada district of India. Lovelock et al.^45^ identified technical issues and over ambitiousness as the major failure of mangrove restoration programmes. They emphasized on the need of identification of suitable land and species for mangrove restoration. Lovelock and Brown^46^ proposed land tenure consideration as one of the important factors for mangrove restoration. Su et al.^47^ conducted a meta-analysis of the outcome of the mangrove restoration from 167 peer-reviewed articles and they stressed on ecological analysis for mangrove restoration. Lee et al.^48^ focused on the evidence-based restoration policies for both short term and long-term restoration programmes.

Many scholars have utilized SDMs for mapping suitable habitats of mangroves species^1,4,24,49–53^. Sarker et al.^24^ prepared spatial density maps of prominent mangrove species and analyzed habitat suitability in Bangladesh Sundarban using generalized additive models (GAMs). Hu et al.^4,51^ used the maximum entropy (MaxEnt) model for assessing the habitat suitability of mangrove forests in China. Rodríguez-Medina et al.^52^ also used MaxEnt model for determining suitable habitat distribution mangroves in Mexico. Wang et al.^53^ used this method for assessing habitat suitability in Guangdong Province of coastal China. Chakraborty et al.^49^ used Analytic Hierarchy Process (AHP) for assessing potential mangrove suitability in the Andaman Islands of the Indian Ocean. All these mangrove habitat suitability studies used ‘a single model fits all' approach. Such an approach may not always provide a complete picture of the habitat distribution due to physiographic differences, species-specific responses to the climate and environmental conditions. To overcome this problem, Banerjee et al.^1^ assessed mangrove habitat suitability for seven true mangroves species over the Indo-West Pacific region using an ensemble of eight different machine learning models. However, the wide resolution being 2.5 arc minutes resolution was the major limitation of this study. Such a resolution cannot provide accurate information on species distribution. Multi-modeling habitat suitability assessment based on fine resolution using species occurrence data can help in protecting the threatened ecosystems. Thus, this is an urgent need to apply the species occurrence and environmental data to setup a finer resolution multi-modelling habitat suitability assessment at regional scale for protecting threatened ecosystems. Furthermore, linking the species distribution models with nature-based restoration of mangrove species is an important research gap. Thus, we have applied the multi-modeling species distribution approach in Indian Sundarban- the largest single block of mangrove species. Our study aims to achieve two specific objectives. Firstly, assessment of multi-modeling habitat suitability for 18 true mangrove species at a finer spatial scale, and secondly, prioritization of species-specific ecological restoration using a grid-based approach.

## Study area

**Figure 1:**
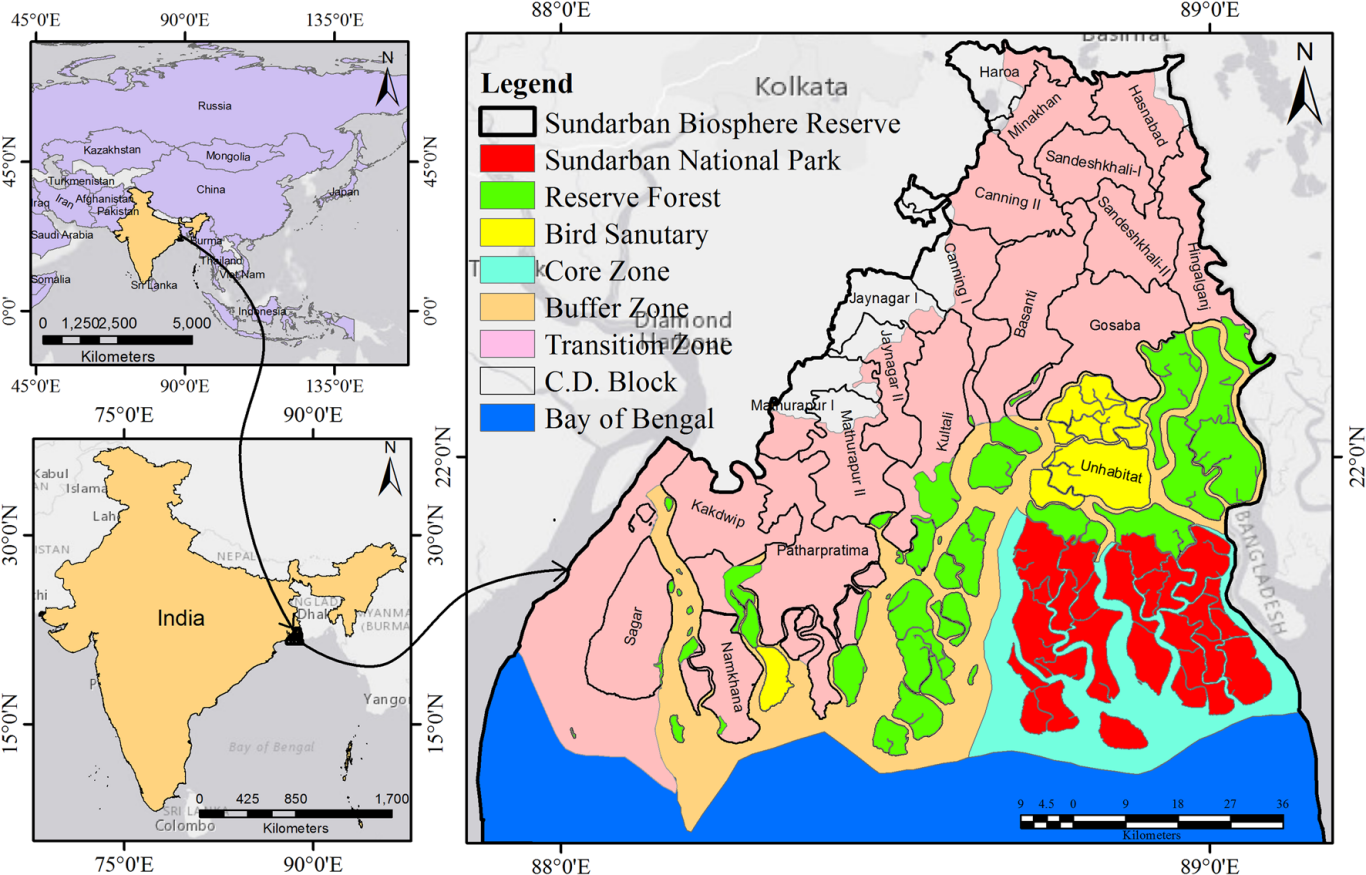
Location of Sundarban Biosphere Reserve in India (The map was prepared using ArcGIS 10.8.2 software [https://desktop.arcgis.com].)

Sundarban Biosphere Reserve (SBR) extends between 21° 31′ N and 22° 30′ N latitudes and 88° 10′ E and 89° 51′ E longitudes (Fig. 1). Of the total geographical area (9630 km^2^), 4266 km^2^ area is under mangrove forest. Of the total islands in the SBR (102), 48 islands are uninhabited and covered by mangrove forests. Most of the Reserve consists of low-lying alluvial mudflats, tidal creeks, and multiple river channels. Sundarban has great mangrove diversity because of its highest number of mangrove plant species and the densest and tallest mangrove forest in the world^54^. It provides the best example of a low-energy wave coast, where mangroves grow luxuriantly along the seashore^55^. Mangrove forests can best be flourished in the estuarine regions where a large amount of freshwater is discharged for a longer duration of time in a year. The richness of the Sundarban mangrove forest depends on the freshwater flow from the River Ganges and the Brahmaputra and its tributaries/distributaries throughout the year. The Sundarban mangrove support sustaining the habitat of the Royal Bengal tiger (Panthera tigris) and Ganges river dolphin (Platanista gangetica). Recently Indian Sundarban has been declared a Ramsar site under the Ramsar Convention in 2020 because of its ecosystem’s services, biological diversity, and universal value^18,55,56^. The total area of Sundarban Biosphere Reserve is divided into the core zone (national park), buffer zone (reserve forest and bird sanctuary and the transition zone (human habitation).

## Methodology

**Figure 2:**
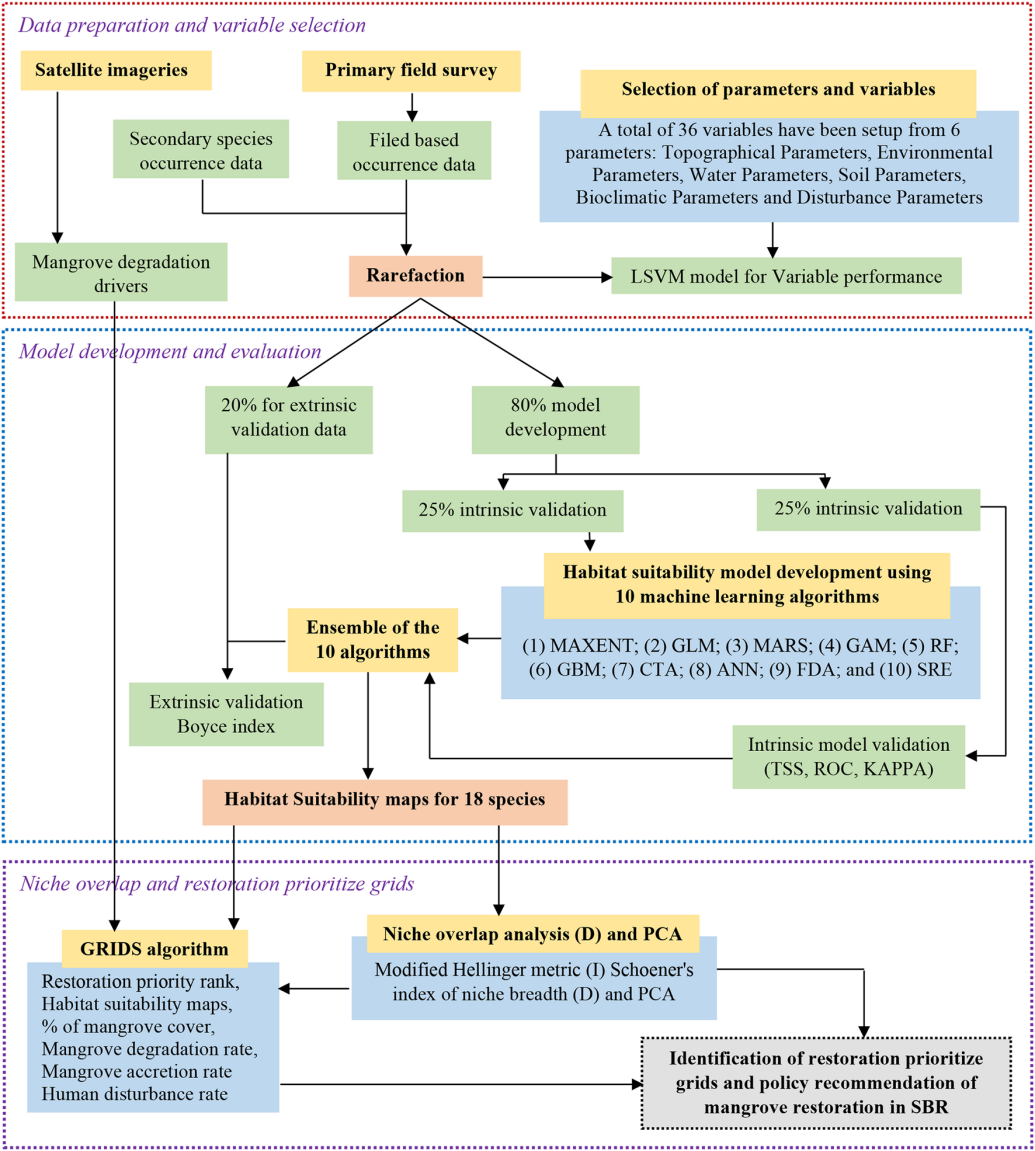
Methodological framework of the study.

**Figure 3:**
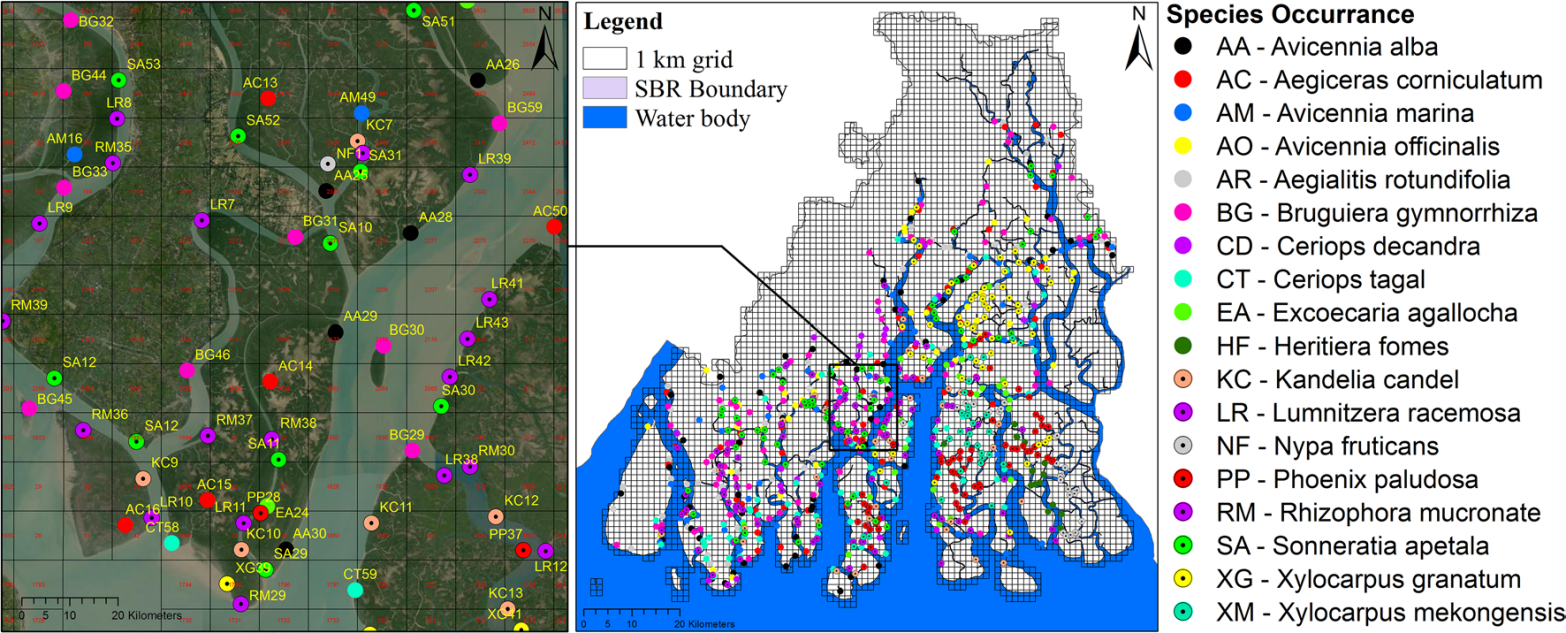
A grid-based field survey (2018–2019) has been conducted to collect the species occurrence data of 18 mangrove species. (The satellite images are obtained from the ArcGIS google earth base map. The species occurrence locations were collected through Garmin eTrex 32 × Outdoor Handheld GPS and the maps were prepared using ArcGIS 10.8.2 software [https://desktop.arcgis.com].)

The present study makes an attempt to prioritize restoration target areas by using mangrove habitat suitability models, niche overlay function, and mangrove degradation rate using a grid-based approach over the Sundarban Biosphere Reserve. Degraded mangrove areas between 1975 and 2020 were identified using Landsat images and field verification of the specific sites. The SBR was divided into 5609 grids using a 1 km grid to understand the nature of mangrove degradation and modeling purpose (Fig. 3). Landsat MSS (1975) and Landsat 8 OLI (2020) satellite data were utilized for preparing a land use/landcover map of the SBR using a supervised classification technique. The classification accuracy was assessed through the confusion matrix and kappa coefficient. Overall accuracy was found 87.62% for 1975 and 94.9% for 2020 and the kappa coefficient values was 0.89 for 1975 and 0.96 for 2020. Post classification change matrix technique was used to prepare land use/land cover change map during 1975 and 2020. The area under vegetation cover and mangrove was extracted from the land use land cover map. NDVI maps during different seasons were utilized to improve the accuracy of the vegetation cover map of the SBR. Ten influencing drivers were selected for analyzing the habitat suitability of mangrove forests (Table 1). The details of the methodological framework are presented in Fig. 2.

**Table 1:** Major drivers of mangrove degradation in Sundarban Biosphere Reserve.

No	Factors	Major drivers	References
1	Deforestation and habitat fragmentation	Nearly 341 sq. km area under mangrove forest has decreased during 1975–2018 due to natural hazards and anthropogenic factors	^57^
2	Natural hazards and extreme weather	The SBR has registered a 26% increase in tropical cyclones in last 120 years. Natural hazards and extreme weather events are the main factors of mangrove degradation	^22^
3	Salinity intrusion	Increase in soil salinity is one of the important problems for mangrove degradation in Sundarban. Intrusion of saline water from Bay of Bengal is the reason for increased of salinity in the SBR. Increase in salinity in the Reserve is due to intrusion of saline water	^58^
4	Sea level rise	The rate of sea level change is higher than global average	^59^
5	Coastal erosion and losses of islands	Southern part of the Reserve is active delta. So, erosion and accretion are important factors for mangrove loss and gain. The average rate of erosion in SBR was found to be 5.98 m^2^/year	^60,18^
6	Construction of earthen embankments	Construction of earthen embank is an important problem for mangrove degradation. The length of the embankments in Sundarbans alone is 3638 km which altered the tidal inundation, sediment accretion and geomorphic character of the deltaic inlets	^61^
7	Aqua-culture/ shrimp farming	In last few decades many agricultural land, mangrove patches and fallow areas has been converted to shrimp farming. These types of conversion have huge impact on small mangrove patches within the upper part of the Reserve	^62,63^
8	Population growth and built-up expansion	Many studies indicate that tremendous increase in built-up area, concreate road, brick kilns has impacted on decrease in forest areas. The Sundarban has 4.37 million population with 975 persons/sq. km population density which is a huge pressure on forest ecosystems	^64,59^
9	Pollutions and industrial waste	A large volume of sewage from the city of Kolkata is drained into East Kolkata Wetland. Industrial pollution from river Hooghly and arsenic pollution have become serious threat to the mangrove ecosystems and habitat	^65^
10	Poverty and backwardness and lack of awareness	Inadequacy of resources, poverty and remoteness are big challenges for the Sundarban community. Nearly 43.5% population lives below poverty line. So due to large scale poverty, local communities very much depend on the forest products. Most of the islands of the SBR have low health and education facilities and inadequate amenities	^66,67^

### Occurrence data

We selected 18 dominant mangrove species in Indian Sundarban for preparing a habitat suitability model (Table 2). The occurrence data of the selected mangrove species have been collected from Global Biodiversity Information Facility (GBIF) and the existing published literature^68,69^. The occurrence data were collected from the published sources^70–74^ and field survey using a grid-based stratified random sampling approach during 2018–2019. We received help from the local fisherman, crab and honey collector to identify mangrove species and collected samples from the core forest areas. The total grids were divided into 19 strata as per the administrative blocks for an easy process and 50 samples were selected from each stratum. In this way, a total of 950 occurrence locations were selected from the field survey (Fig. 3). The secondary occurrence data was verified through google earth images to avoid duplication. Further, the occurrence data was run through a statistical assessment using spatially rarefied in ArcGIS SDM toolbox by selecting a single point grid cell for individual species (1 km grid). Finally, we selected a total of 1560 occurrence data (707 from the archive sources and 853 from the field survey (Table 2) to develop species distribution models.

**Table 2:** Details of the selected mangrove species and the occurrence data.

Sl no	Local name	Code	Scientific name	Family	IUCN status	Occurrence data	GPS verified points	Inter tidal position
1	Tora	AR	Aegialitis rotundifolia	Plumbaginaceae	Near threatened	35	60	Low
2	Khalsi	AC	Aegiceras corniculatum	Myrsinaceae	Least concern	34	55	Middle
3	Kalo Baine	AA	Avicennia alba	Acanthaceae	Least concern	45	60	Low
4	Peara Baine	AM	Avicennia marina	Acanthaceae	Least concern	56	60	Middle
5	Jat Baine	AO	Avicennia officinalis	Acanthaceae	Least concern	65	40	Middle
6	Bakul Kankra/ Champa	BG	Bruguiera gymnorrhiza	Rhizophoraceae	Least concern	64	80	Low
7	Jhamti Garan/ Jele Garan	CD	Ceriops decandra	Rhizophoraceae	Near threatened	50	30	Low
8	Jat Garan	CT	Ceriops tagal	Rhizophoraceae	Least concern	38	65	Low
9	Genwa	EA	Excoecaria agallocha	Euphorbiaceae	Least concern	25	25	Middle, high
10	Sundari	HF	Heritiera fomes	Malvaceae	Endangered	10	15	Middle, high
11	Goria	KC	Kandelia candel	Rhizophoraceae	Least concern	23	35	Middle, high
12	Kripa/ Kripal	LR	Lumnitzera racemosa	Combretaceae	Least concern	40	45	Middle
13	Gol Pata	NF	Nypa fruticans	Arecaceae	Least concern	25	25	High
14	Hental	PP	Phoenix paludosa	Arecaceae	Near threatened	36	60	Middle, High
15	Garjan/ Bhara	RM	Rhizophora mucronate	Rhizophoraceae	Least concern	45	47	Middle
16	Tak Keora	SA	Sonneratia apetala	Lythraceae	Least concern	60	66	Middle
17	Dhundul	XG	Xylocarpus granatum	Meliaceae	Least concern	36	55	Middle, high
18	Pashur	XM	Xylocarpus mekongensis	Meliaceae	Near threatened	20	30	Middle, high

The species code has been prepared using the first letter of the species, hereafter this species code has been used throughout the manuscript. The species name and family name has been standardized through IUCN species list and the World Flora Online^75^. The species occurrence point data has been collected from different sources like the Global Biodiversity Information Facility (GBIF) database and literature. The GPS survey was carried out to collect the maximum possible occurrence data from the field. The information about the common local habitat of the selected species was confirmed through the local people and the literature.

### Data preparation and variable selection

After a detailed literature review and field knowledge, we considered 36 variables from 6 different parameters for the present study. The data source, data structure, spatial resolution, and references of the data have been provided in Appendix 1. All the raster variables were re-sampled into 1 km resolution using resample tool in ArcGIS to match the original resolution of WorldClim data and the 1 km sampling grid^76^. All 30-m raster images were reclassified into 1 km grid cell and the average number of pixels over 5609 girds in Sundarban were determined to be 33,334. From the topographical parameters, we selected six important variables (Appendix 2A) namely, elevation, slope, curvature, geomorphology, topographic wetness index (TWI), and stream power index (SPI) (Appendix 1). All the selected topographical variables were intricately linked with the tidal inundation and temperature, which can directly influence the distribution of mangrove species^77^. From environmental parameters, 6 variables (Appendix 2B) were selected namely normalized difference vegetation index (NDVI), level of flood inundation, mean tidal range, storm surge height, normalized difference moisture index (NDMI), and rate of erosion/accretion (Appendix 1). Mangroves are known as salt-tolerant higher groups of coastal plants^78^ which are mainly flourish in the backwater, estuaries, creeks, and lagoons. These flowering plants are inundated and exposed two times a day during high tide and low tide with coastal marine water and grow up by the mixture of the freshwater flow from upstream. Thus, water is a very important parameter for assessing mangrove habitat suitability. Six variables were selected as underwater parameters namely water surface temperature, water salinity, PH value, distance from water, drainage density, and modified normalized difference water index (Appendix 2C). All these variables are directly associated with the growth and distribution of mangrove species in Sundarban (Appendix 1). Soil is considered an influencing parameter along with climate for the distribution of flora at spatial scales^1,79^. However, few studies have considered soil variables in modeling mangrove distribution^52^. Six important variables namely soil texture, soil salinity index, soil fertility index, sediment yield factor (Ton), electronic conductivity (EC), and vegetation soil salinity index (VSSI) were selected to examine the influence of soil (Appendix 2D).

Bioclimatic layers are one of the popular variables widely used by various scholars in order to build species distribution and ecological niche modeling^80^. Many researchers have also used the bioclimatic variables for mangrove habitat suitability models because of their association with climate-related phenomena, especially temperature and precipitation^81^. Principle component analysis (PCA) was performed to remove the data redundancy and avoid the random error and uncertainty of the model (Appendix 3). Of the 19 variables only the most suitable 6 bioclimatic variable layers were used for this study: BIO1, Annual Mean Temperature; BIO2, Mean Diurnal Range; BIO3,  Isothermality; BIO4, Temperature Seasonality, BIO12 = Annual Precipitation and BIO15 = Precipitation Seasonality (Appendix 2E) Anthropogenic activities are the main factors for the decrease in areas under mangroves globally^82^, Sundarban is no exception to this encroachment. Six disturbance variables namely distance from the road, road density, distance from Settlement, population density, distance from agriculture field, and embankment density were selected for habitat suitability analysis based on local knowledge (Appendix 2F).

### Variable performance for habitat suitability model

Model collinearity among the variables was checked to avoid the negative influence of variables on the performance of the model. The predictive ability of all individual variables was tested before performing the suitability model^83^. The linear support vector machine (LSVM) model was utilized for assessing the prediction capacity of variables. LSVM can be expressed as:

$$g(x)=Sgn({w}^{T}a+b)$$

where, ${w}^{T}$ is the weight matrix of the habitat suitability variables, $a=$ (a_1_, a_2_…a_14_) vector inputs of the variables, $b$ is the offset from the origin of the hyper-plane. The variables *i*th with the weight ${w}_{i}$ close to 0 has a smaller effect on the prediction than the one with larger values of ${w}_{i}$. The average merit (AM) for each variable and its ability for prediction ranged between 1 and 10 (Table 3). The values > 5 represent the potential influencing variables for habitat suitability model.

**Table 3:** Intrinsic and extrinsic model evaluation scores for each species.

No	Species	Intrinsic			Extrinsic
		ROC	TSS	KAPPA	Boyce Index
1	Aegialitis rotundifolia	0.923	0.914	0.936	0.874
2	Aegiceras corniculatum	0.895	0.901	0.914	0.841
3	Avicennia alba	0.856	0.914	0.987	0.879
4	Avicennia marina	0.954	0.939	0.964	0.903
5	Avicennia officinalis	0.865	0.996	0.967	0.988
6	Bruguiera gymnorrhiza	0.821	0.761	0.988	0.859
7	Ceriops decandra	0.854	0.981	0.787	0.852
8	Ceriops tagal	0.882	0.978	0.932	0.847
9	Excoecaria agallocha	0.987	0.987	0.852	0.895
10	Heritiera fomes	0.862	0.869	0.847	0.866
11	Kandelia candel	0.889	0.941	0.873	0.888
12	Lumnitzera racemosa	0.841	0.789	0.896	0.989
13	Nypa fruticans	0.832	0.919	0.853	0.788
14	Phoenix paludosa	0.943	0.928	0.908	0.987
15	Rhizophora mucronate	0.938	0.976	0.909	0.967
16	Sonneratia apetala	0.994	0.806	0.994	0.969
17	Xylocarpus granatum	0.955	0.821	0.828	0.998
18	Xylocarpus mekongensis	0.951	0.842	0.847	0.906
Average (± SE)		0.902 (0.018)	0.904 (0.017)	0.905 (0.013)	0.905 (0.011)

### Model development and validation

The habitat suitability models for 18 selected mangrove species were constructed using ten algorithms through the biomod2 package in R studio. Machine learning algorithms and statistical models can be set up in the R studio package for getting different complex conditions and properties which can give a possible higher accuracy on the species distribution model^84^. For the present study, we performed 10-machine learning algorithms: (1) MAXENT: Maximum Entropy; (2) GLM: Generalized Linear Model; (3) MARS: Multiple Adaptive Regression Splines; (4) GAM: Generalized Additive Model; (5) RF: Random Forest; (6) GBM: Boosted Regression Trees model; (7) CTA: Classification Tree Analysis, (8) ANN: Artificial Neural Network; (9) FDA: Flexible Discriminant Analysis and (10) SRE: Surface Range Envelop model. The performance of all ten models has been assessed through the intrinsic model building process and extrinsic validation methods. For this, we have split the occurrence data into 80% for training and 20% for testing the model. For creating the intrinsic model, we randomly used 75% of the occurrence data for training the model and the remaining 25% for testing each individual model. The modeling process was run for 18 different species using 10 machine learning algorithms. In this way, 180 models were run. Cohen's Kappa (KAPPA), true skill statistic (TSS), and the receiver operating characteristics (ROC) models were used for validated all the individual models.

Finally, for the extrinsic evaluation, the algorithms which have > 0.75 predicting performance for all the validation models (KAPPA, TSS, and ROC) were used to build the final ensemble model (Appendix 4). We have used the ensemble forecast method using a weighted mean approach in biomod2 packages within R studio for producing the final habitat suitability models for all the selected species. The final habitat suitability models for all 18 species were again validated through the Boyce index (BI) using the 20% data which were kept aside for validation^85^ (Table 3). The output habitat suitability raster index from the ensemble models for all species was then categorized into four classes namely highly suitable, moderately suitable, slightly suitable, and not suitable.

### Niche overlap analysis and setting restoration prioritization

Modified Hellinger metric (I) and Schoener's index of niche breadth (D) were used to estimate the niche overlap among 18 species. The spatial similarity and dissimilarity of niche area for the selected species has been calculate using the raster calculator spatial overlay function in ArcGIS.

We have applied a principal component analysis (PCA) to prepare a restoration priority rank for all 18 species based on their niche characteristics, estimated potential distribution, local knowledge, and degradation status.

### Identification of restoration prioritize grids

Percentage of niche overlap data, restoration priority rank for all species, habitat suitability maps for all individual species, percentage of mangrove cover, mangrove degradation rate, mangrove accretion rate, and human disturbance rate were used as input functions for identifying the restoration prioritized grids over 5609 girds in Sundarban. Grid-wise total mangrove area for the present scenario was calculated from the land use/land cover map for 2020. A grid-wise mangrove degradation rate and mangrove accretion rate have been calculated using the land use land cover change between 1975 and 2020. The human disturbance rate was calculated using the following equation:

$$\mathrm{HDR}=\frac{(BR+NP+RD+AR)}{1km}*100$$

where HDR is the human disturbance rate, BR is the percentage of built-up area in each grid, NP is the number of populations in each grid, RD = road density in each grid and AR is the percentage of agricultural area in each grid.

For the preparation of final restoration prioritize grids we developed a species-wise grid-based algorithm using the FactomineR package in R studio. The final output of the species level restoration prioritize grids were assessed by using negative and positive indicators function. The negative indicators included: (1) > 80% present mangrove cover grid in 2020, (2) HDR value more than > 75%, (3) not suitable and slightly suitable grids, (4) natural accretion rate as > 75%. The positive indicators for this algorithm involved: (1) restoration priority rank for each individual species, (2) niche overlap grid for each individual species (P < 0.05), (3) highly and moderately suitability grids for each individual species, (4) degradation date > 75%.

## Results

### Mangrove degradation in SBR

**Figure 4:**
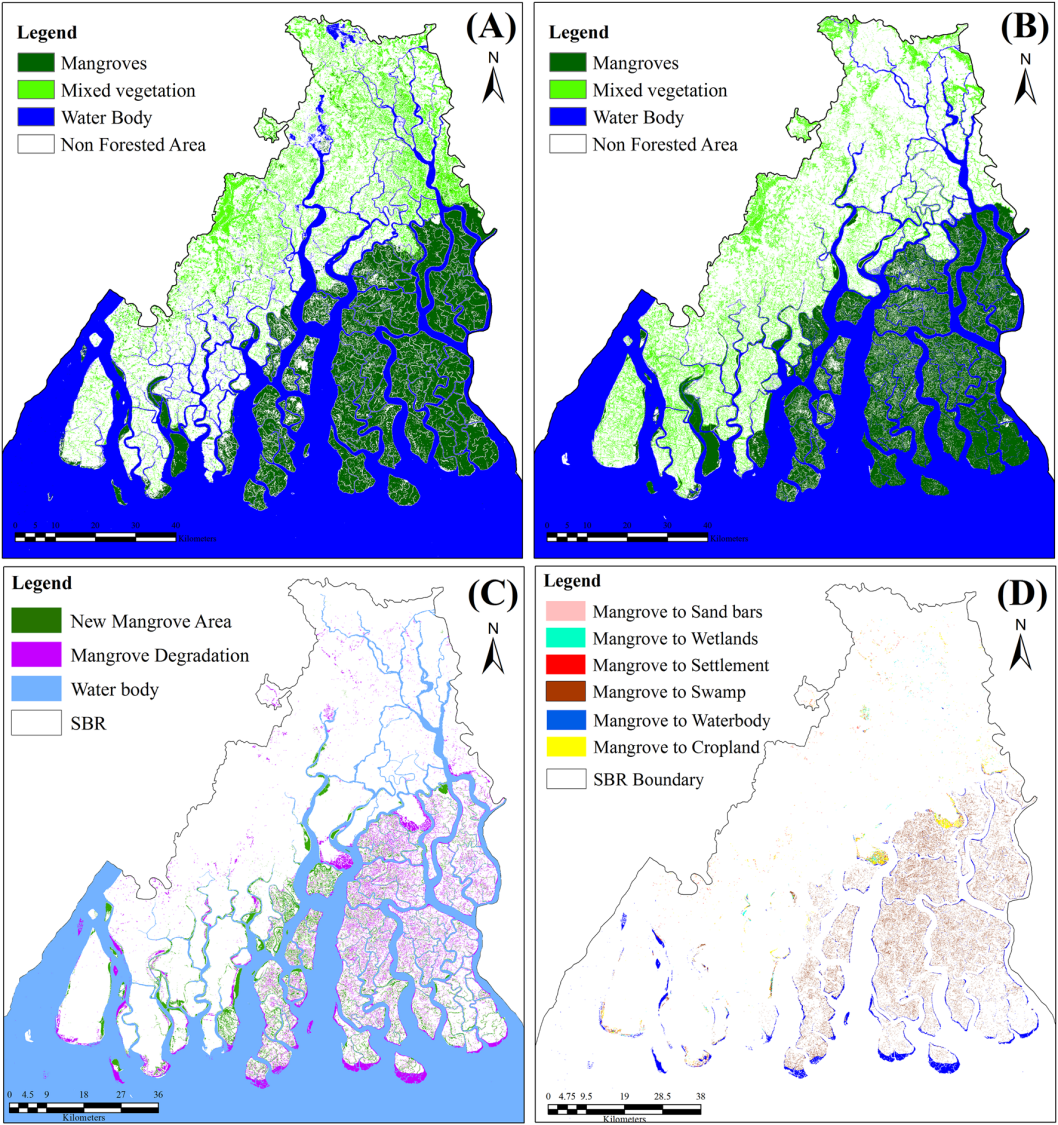
Mangrove cover map of Sundarban Biosphere Reserve during 1975 (**A**) and 2020 (**B**), Mangrove accretion and mangrove degradation over 1975–2020 (**C**) and Nature of mangrove degradation in Sundarban Biosphere Reserve (**D**). (The Landsat satellite images are obtained from the EarthExplorer [https://earthexplorer.usgs.gov/]. The maps were prepared using ERDAS IMAGINE 2014 [https://hexagon.com/products/erdas-imagine] and ArcGIS 10.8.2 software [https://desktop.arcgis.com].)

**Figure 5:**
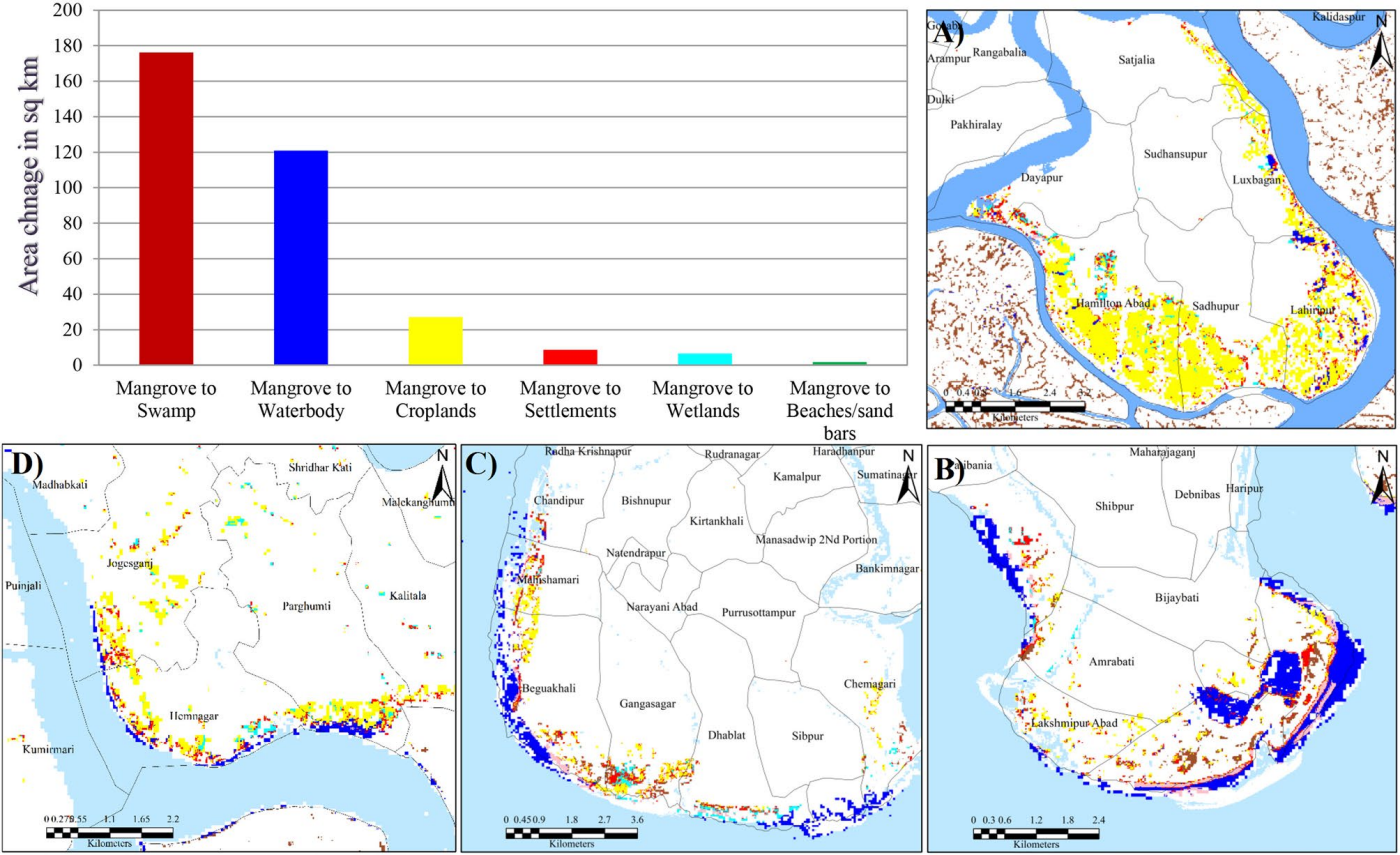
Change of mangrove cover under different land use/land cover classes Four important degraded mangrove hotspots in SBR; (**A**) Dayapur and Annpur village of Gosaba block mainly due the agricultural encroachment, (**B**) major built-up expansion Bakkhali area due the tourism development and coastal erosion at, (**C**) beaches and sand bar expansion at southern part of Sagar Island, (**D**) waterlogging wetlands and agricultural encroachment at Southern part of Hingalganj.). (The Landsat satellite images are obtained from the EarthExplorer [https://earthexplorer.usgs.gov/]. The maps were prepared using ERDAS IMAGINE 2014 [https://hexagon.com/products/erdas-imagine] and ArcGIS 10.8.2 software [https://desktop.arcgis.com].)

The land use land cover change analysis revealed that the Sundarban mangrove forest has decreased by nearly 341 sq. km from 1975 to 2020 mainly due to anthropogenic encroachments, geomorphic processes, and climate change-induced hazards. The area under mangrove has transformed into swamps (176 km^2^) followed by water bodies (121 km^2^), settlements 8.6 km^2^), wetlands (6.5 km^2^), and sand bars (1.7 km^2^). The main causes of the degradation included sea-level rise, sudden disaster events, over-exploitation, and lack of sustainable adaptative strategies. The area under mangroves in the Reserve has decreased from 22.9% in 1975 to 19.8% in 2020 (Fig. 4, Appendix 5). As Sundarbans is a dynamic active delta, thus natural regeneration of new mangrove and mangrove degradation occurred together but the rate of mangrove degradation is much higher than the natural regeneration. The regeneration of new mangroves was mostly observed in the new char islands due to the depositional process (Fig. 5). The regeneration of the new mangrove rate has been increased after 1990 due to the new land reformation and conservation laws implemented by the government.

**Figure 6:**
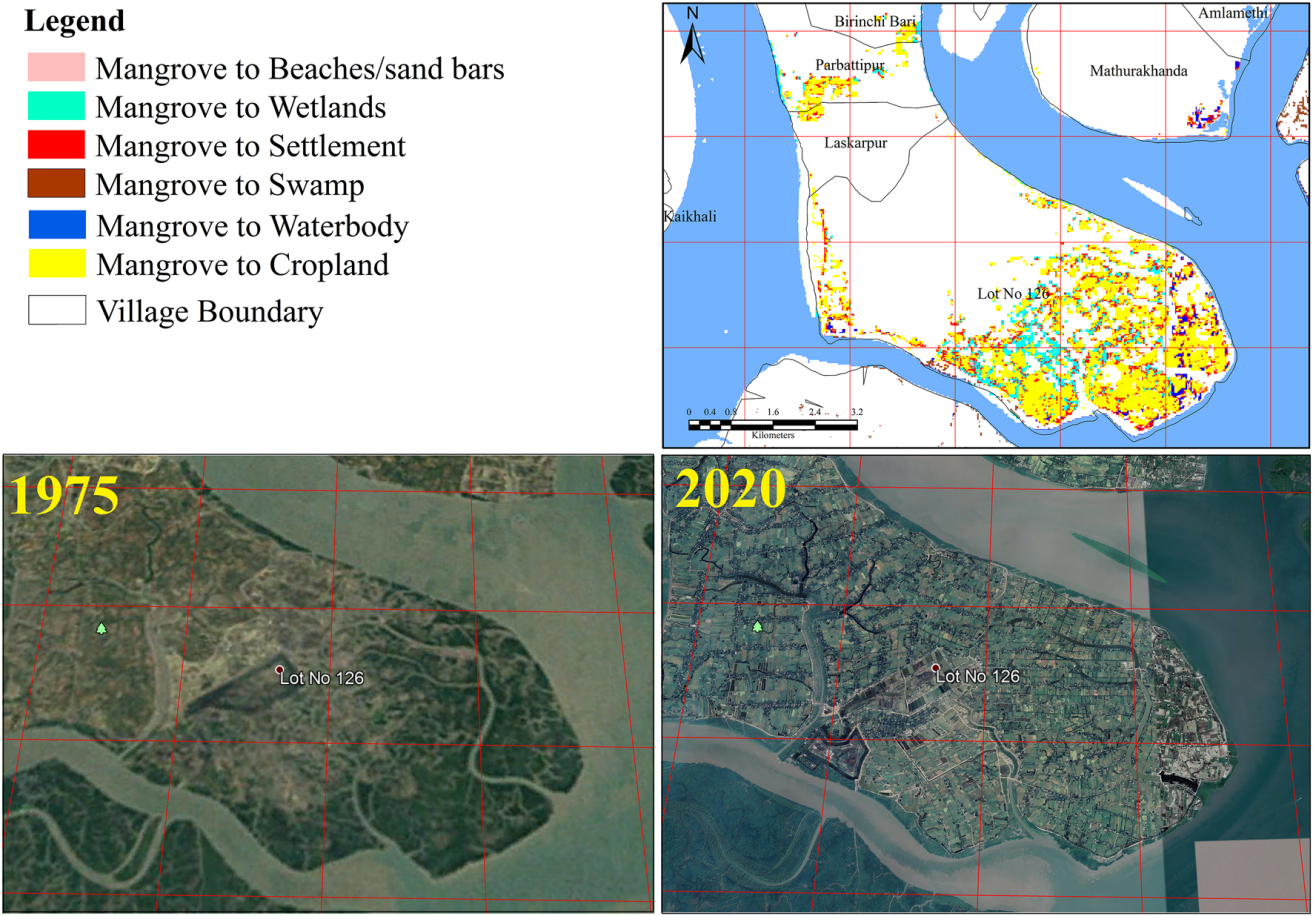
Deforested mangrove areas due to resettlement of partition refugees and recently tourism development (google earth images and LULC change map 1975–2020). (The satellite images are obtained from the google earth pro software [https://www.google.com/intl/en_uk/earth/about/versions/]; version 7.3 and EarthExplorer [https://earthexplorer.usgs.gov/]. The maps were prepared using ERDAS IMAGINE 2014 [https://hexagon.com/products/erdas-imagine] and ArcGIS 10.8.2 software [https://desktop.arcgis.com].)

The analysis here shows that the degradation of mangroves occurring in the SBR is due to both anthropogenic and natural factors. It has been observed that about 85% area under mangroves has been converted to sand bars, wetlands, swamps, settlements, cropland, and water body. It signifies that major mangrove degradation in the SBR is due to geomorphological, hydrological, and climatic factors. However, the analysis shows that the remnant 15% area under mangroves is under degradation due to anthropogenic activities. This percentage is likely to increase more in the future with the continuous rise in the population and increasing pressure on the already existing land resources (Fig. 6). Thus, anthropogenic activities need to be checked for better management of mangroves in the region.

Mitigation with respect to climatic factors responsible for degradation requires a significant long-term approach. However, when we consider mitigating the anthropogenic factors, it is still very much under control. A slight modification in our approach can lead to utilizing these resources to manage and conserve the rich mangrove ecosystem in this region. Most of the area under mangrove forest in the SBR is facing the threat of degradation either from natural factors or from human encroachment^86^. Certain pockets in the SBR have become the hotspots of mangrove degradation.

### Contribution of variables for habitat suitability models

Linear support vector machine (LSVM) model-based variable performance analysis revealed that the variables with > 5.0 average have the maximum influence on mangrove habitat suitability models in the SBR (Table 4). Six bioclimatic variables have a high contribution to the habitat suitability for most of the species. Four variables from disturbance indicators have a higher contribution to the habitat suitability models namely distance from Settlement, population density, distance from crop field, and embankment density. The only slope has performed well from the topographical variables. NDVI, mean tidal range and rate of erosion/accretion among the environmental parameters were found influencing variables for habitat suitability. Distance from water and drainage density among water parameters and only soil salinity index variable from soil parameter were found good performance variables. However, the contribution of these variables varied among species.

**Table 4:**
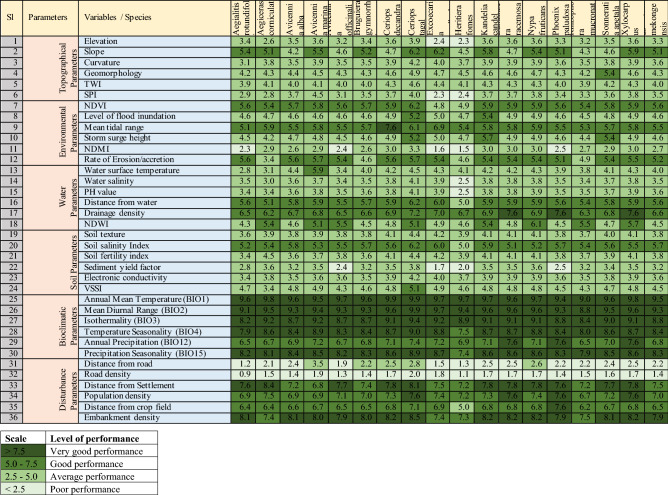
Contribution of the individual layers to the habitat suitability models (average merit (0–10), > 5.0 is good performance).

### Potential distribution of mangrove species

**Figure 7:**
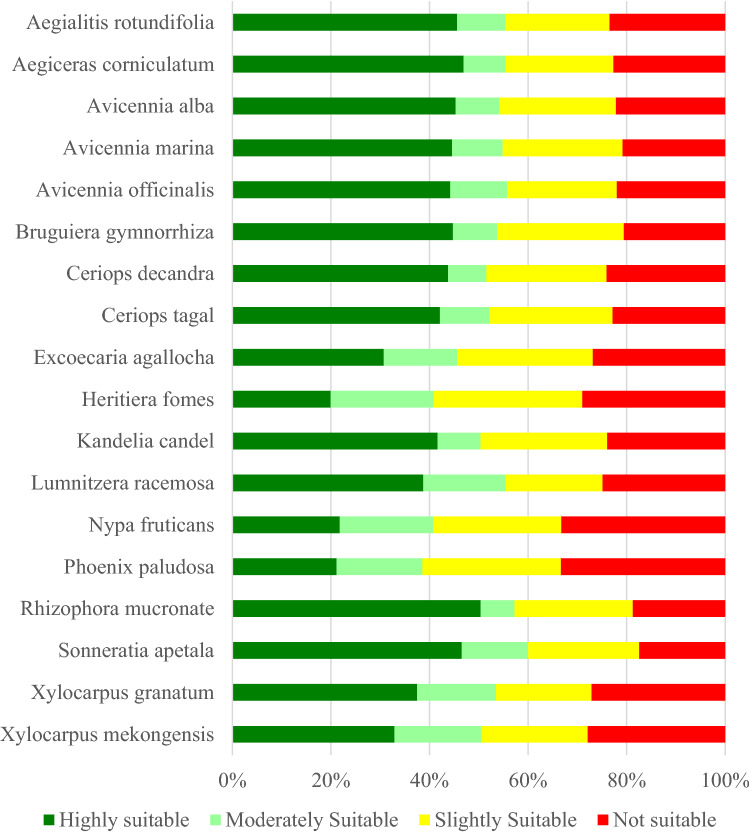
Species-wise percentage of area under different habitat suitability classes.

**Figure 8:**
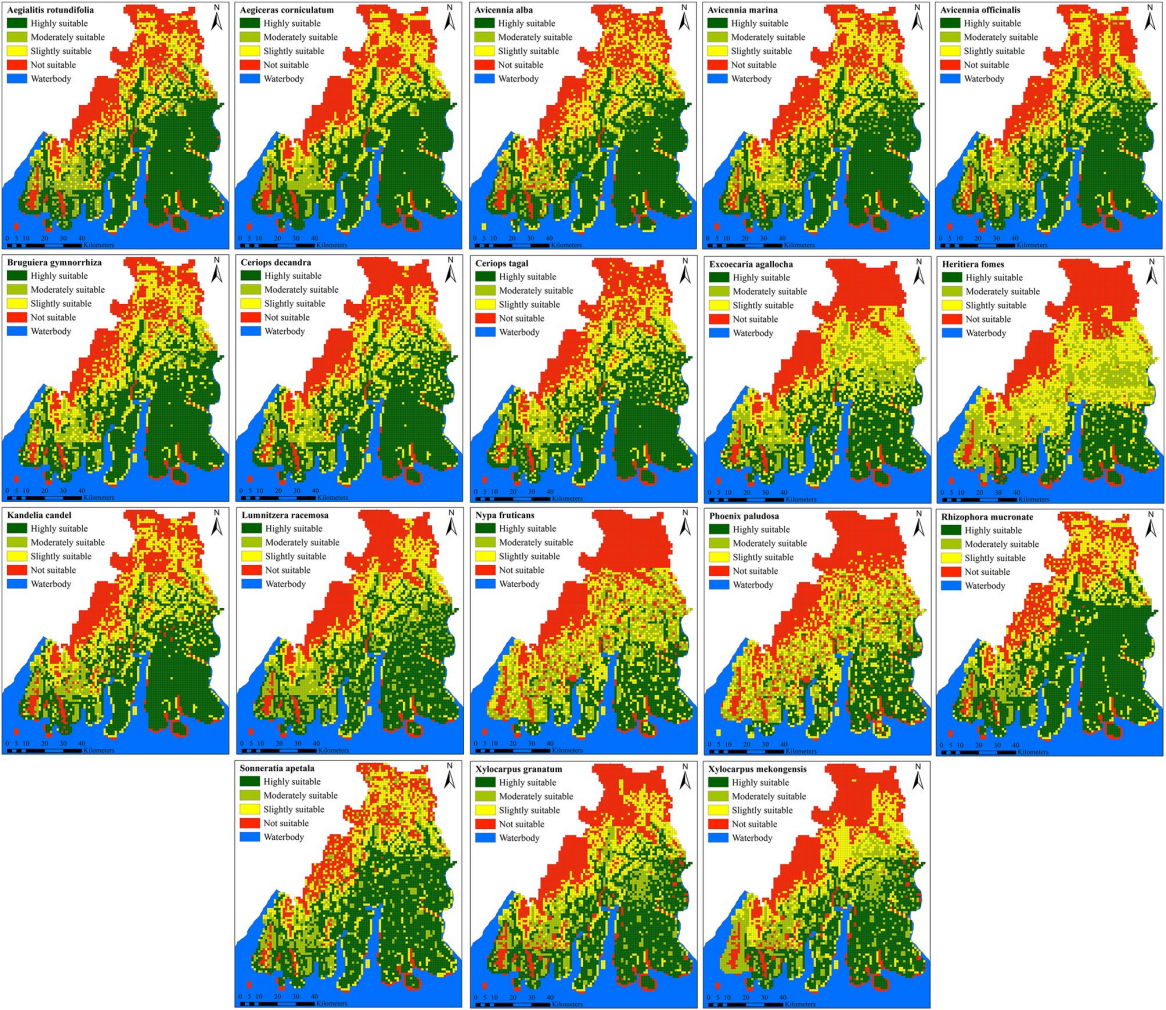
Habitat suitability maps for the selected 18 true mangrove species in the Sundarban Biosphere Reserve The maps were prepared using ArcGIS 10.8.2 software [https://desktop.arcgis.com].)

The final ensemble models-based habitat suitability maps for all species were categorized into four suitability classes namely highly suitable, moderately suitable, slightly suitable, and not suitable girds. The habitat suitability models revealed that the largest area of the SBR was found under highly suitable habitats (36.6%) followed by slightly suitable (32%), not suitable (16.5%), and moderately suitable (13%) (Fig. 7). The spatial distribution of the habitat suitability analysis revealed that northern and north-western parts of the Reserve were found under the not suitable category for most of the species due to human disturbance and their location away from the actual coast (Fig. 8). The individual species-level analysis also revealed that highly suitable habitats for rare and endangered species like *Heritiera fomes, Phoenix paludosa, Nypa fruticans and Xylocarpus mekongensis* were found in the core and buffer zone of the Sundarban which are situated within the uninhabited islands. Highly suitable habitats for the species like *Bruguiera gymnorrhiza, Avicennia alba, Aegialitis rotundifolia, Sonneratia apetala, Aegiceras corniculatum, Rhizophora mucronate* were also found far away from the actual coast. These species were distributed in human habitats. Further, low intertidal position species like *Bruguiera gymnorrhiza, Ceriops decandra, Ceriops tagal, Avicennia alba and Aegialitis rotundifolia* were commonly found in the buffer and transitional zones of the Reserve (Fig. 7, Appendix 6).

### Niche overlap and validation of models

The principal component analysis revealed that all the selected species have a specific niche area within the Reserve. Very high niche overlap was identified mostly for the same family species and sometimes for the different family species also. The grid-based spatial similarity and dissimilarity analysis revealed that the highest species level niche overlap has been found within the uninhabited areas where the species varieties are maximum within the grids. Low niche overlap among all species was found in the areas located away from the coast. The areas located near the river bank were also found to have high to moderate level spatially niche overlap (Table 5).

**Table 5:**
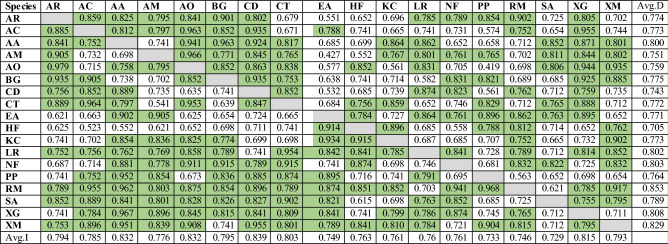
Estimates of niche overlap between selected species: upper diagonals: Schoener’s index of niche breadth (D); lower diagonals: modified Hellinger metric (I).

The green colour indicate significance at p < 0.05 level.

### Restoration prioritization

**Figure 9:**
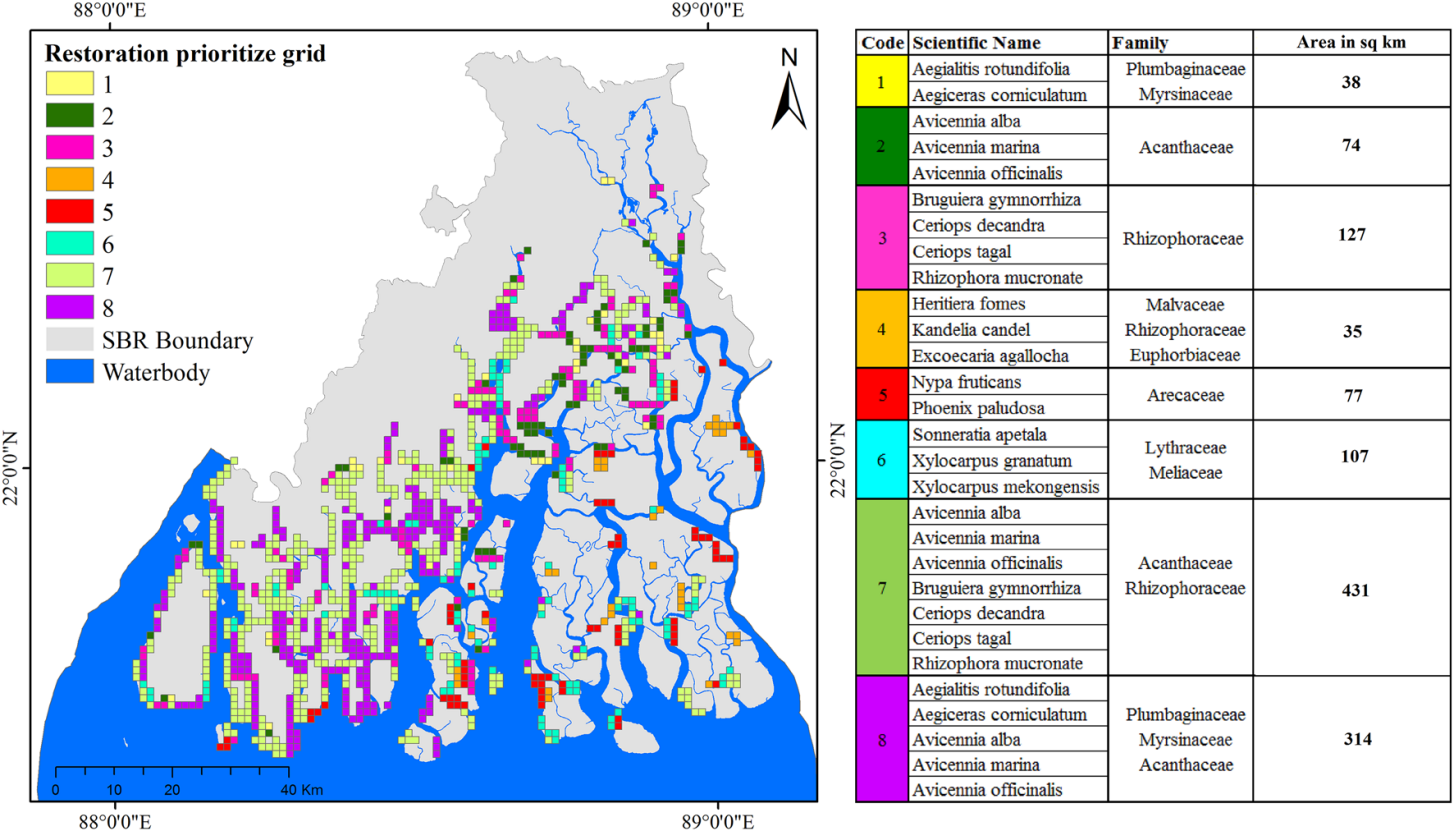
Species wise restoration prioritize grids for 18 selected mangrove species (The map were prepared using ArcGIS 10.8.2 software [https://desktop.arcgis.com].)

The grid-based restoration prioritization analysis revealed that out of 5609 grids, 1203 grids were found restoration prioritized grids. Further, restoration prioritized grids were categorized into 8 specific prioritization categories. In the first category, two species namely *Aegialitis rotundifolia* and *Aegiceras corniculatum* from *Plumbaginaceae* and *Myrsinaceae* found highly suitable species for restoration prioritization covering an area of over 38 km^2^ in the SBR (Fig. 9). The spatial distribution of these restoration prioritized areas showed that nearly 13% area was found under the uninhabited islands and over 87% area was found under the human habitat islands. In the second category, *Avicennia alba*, *Avicennia marina* and *Avicennia officinalis* from *Acanthaceae* family were found highly suitable species and covered an area of 74 km^2^ in the SBR. In the third category, four species from *Rhizophoraceae* family namely *Bruguiera gymnorrhiza, Ceriops decandra, Ceriops tagal,* and Rhizophora mucronate were found highly suitable species and covered a 127 km^2^ area. Again, for this category, 85% of restoration prioritized areas were found under the habitat islands. In the fourth category, the restoration prioritized areas included the endangered mangrove species in Sundarban namely *Heritiera fomes, Kandelia candel* and *Excoecaria agallocha* covering 35 km^2^ areas in the core forest. The fifth category of the restoration prioritized areas included *Nypa fruticans* and *Phoenix paludosa* species that come under *Arecaceae* family which are also rare and mostly found in the core forest areas. This category covered 77 km^2^ restoration possible areas within the uninhabited islands and most of these areas are located on the edge of the islands as these species are high intertidal species. Three important species namely *Sonneratia apetala*, *Xylocarpus granatum* and *Xylocarpus mekongensis* (Near Threatened) within *Lythraceae* and *Meliaceae* family were included in the sixth category of prioritization. These species were found within the core dense forest areas and covered 107 km^2^ restoration possible area within the core forest and edge areas of the uninhabited islands (Fig. 9).

Mixed species were put under the seventh category of restoration prioritized areas. Under this category, *Avicennia alba, Avicennia marina, Avicennia officinalis, Bruguiera gymnorrhiza, Ceriops decandra, Ceriops tagal and Rhizophora mucronate* were prioritized for restoration. This category covered the highest number of possible restoration areas over 431 km^2^ area. Most of these areas are located on inhabited islands. These restoration prioritized areas are highly associated with the recent mangrove degradation due to anthropogenic and natural causes. The eight categories of restoration prioritized areas were mainly based on the most common species found in the SBR (*Aegialitis rotundifolia, Aegiceras corniculatum, Avicennia alba, Avicennia marina* and *Avicennia officinalis*) and covered an area of 314 km^2^ within the Inhabitat islands. The selected species for this category are commonly used by the local people for their basic needs. This kind of restoration can be helpful for the development and regeneration of community forests which can help to reduce pressure on the core and dense forest areas (Appendix 7).

## Discussion

Mangroves are threatened ecosystems globally due to their coastal location and extensive resources utilized by humans for their needs. The Sundarban mangrove is the largest single block of mangrove in the world and is highly threatened and drastically reducing at an alarming rate due to the overexploitation of resources, land transformation for aquaculture practice, increase in paddy cultivation, infrastructural development, and human settlements.

Climate change-induced coastal hazards and sea-level rise are acting as a significant challenge for mangrove species inhabited in the coastal ecosystems. Such ecosystems are facing habitat degradation due to unprecedented changes in annual mean temperature and precipitation cycle. Thus, habitat suitability modeling can effectively be utilized for conservation planning^87^. However, a lack of understanding of the mangrove distribution and non-availability of occurrence data is an important challenge^88,89^ for habitat suitability modeling. To overcome this problem and to achieve maximum acceptable habitat modeling the present study developed comprehensive field-based occurrence data for 18 mangrove species for conservation and management of mangrove ecosystems in Indian Sundarbans. The present study has adopted a 1 km GRID approach to maintaining consistency in the modeling for each stage to prepare the restoration prioritized grids for individual species. Selection of the habitat suitability variables is an important and crucial task for the construction of the species distribution models^36^. Our result revealed that bioclimatic parameters and disturbance parameters proved to influencing parameters for analyzing habitat suitability in the SBR. Precipitation was found more influencing variable than temperature. The finding is in line with the observation made by a few scholars on a global scale^1,90^ and few are at regional scale^91,92^. The slope was found the most important variable among the environmental parameter for mangrove habitat suitability. Hu et al.^4,51^ and Banerjee et al.^1^ found elevation an important variable. The human disturbance was also found influencing variables for mangrove habitat suitability assessment. The finding is in tune with Banerjee et al.^1^, Hu et al.^4,51^ and Srivastava et al.^93^.

The soil salinity has been significantly negatively correlated with the dominant species habitat such as *Avicennia alba, Avicennia marina and Avicennia officinalis*. Spatio-temporal variation of the salinity adversely affected the mangrove species distribution and composition. Extreme salt stress and high-level salinity affected the growth and structural development of mangrove species in Sundarban^94,95^. A decrease in are under *Heritiera fomes* (endangered) habitat is attributed to increased salinity in the upper part of the Reserve^96^. Intertidal distribution and spatial location of the Sundarban mangrove is also important for species conservation and restoration strategies such as *Heritiera fomes andPhoenix paludosa* in the interior part of the delta, *Excoecaria agallocha* along the riverbank while *Nypa fruticans, Bruguiera gymnorhiza*, *Ceriops tagal* in the moderate location of delta and *Xylocarpus varieties* and *Aegiceras corniculatum* in the more interior parts^97^. The buffer zone of the Sundarban has experienced a high level of deforestation, high level of coastal erosion, high human disturbance, and niche mismatch among the mangrove species. Shortage of freshwater in follow from the upstream, changes in temperature and precipitation pattern caused the habitat shifting and niche mismatching between same family species in the Sundarban. Therefore, the areas that are highly suitable but do not currently have extensive mangrove forests may be locations where mangroves had been previously deforested. These areas could be potential habitats for mangroves. Further, low niche overlap and moderate suitability of maximum species in the eastern part of the Reserve indicated low freshwater supply in the eastern tributaries and streams due to the construction of Farakka Barrage in Murshidabad and thus caused heavy siltation within the creaks and internal channels^98^. Thus, a decrease in freshwater supply could lead to an increase in salinity and push the endangered and threatened species over the edge of abrogation. The increased rate of tropical cyclones during the last few decades has hugely impacted the mangrove density and created a canopy gap in the core forest areas^99^.

Sundarban is one of the most densely populated deltas in the world. A high population has created a huge threat to the species. More than 85% of people in this biosphere region are directly or indirectly dependent on the mangrove ecosystem^55^. The species like *Avicennia alba, Avicennia marina, Avicennia officinalis* and *Rhizophora mucronate* are being threatened due to overexploitation^100^. Thus, the forest dependency of the local people needs to be decreased. Introduction of mangrove social forestry, stakeholder commitment, ecosystems-based community development is required for sustainable mangrove management^101,102^. Further, urban expansion is divested threat to the mangrove ecosystem globally. In Brazil, more than 30% area under mangroves has been destroyed due to urbanization^103^. In Honduras, 9.1% area under mangroves has been destroyed^104^. Urban expansion has greatly impacted the mangrove trees in Mumbai city^105^. Expansion of Kolkata metropolitan areas towards the southwards and many new megaprojects like Joka urban development, a new airport in Bhangar, the rapid growth of Baruipur and Canning are alarming threats to the Sundarban mangrove ecosystem^106,107^. In spite of various laws promulgated by the government for restricting the encroachment of mangrove habitats, this is still being made in the buffer and core forest areas. So, identified restoration prioritized areas can help in the sustainable management and restoration of mangroves in Indian Sundarban.

Our species distribution models and the restoration prioritized locations are based on the 1 km grids-based model output which may not be effective for the real-world implication of restoration programme for few cases. For example, 100% area under all the recommended grids may not be restored due to the real-world shape and other limitations. In our species distribution models, bioclimatic and the environmental factors have played an important role habitat suitability analysis, thus, major uncertainties can be distinguished for the raster binary data of other parameters. Though, biases and uncertainties for the SDM outputs can vary with species and different models used in this study may further improve different grain size output of SDM models. The northern part of the Biosphere Reserve has very spare mangrove patches mixed with other forest areas and this may affect the result of the habitat distribution models. Due to the restriction in the core forest area under the Sundarban national park, we have collected species samples only in the edge of the delta and this may be a limitation for SDM models for few species. Our ecological niche overlay analysis is based on the habitat suitability models and we did not carry out further field verification for validating the niche overlay assessment.

## Policy recommendations for conservation and restoration

Sundarban is a heterogeneous region and several physical and anthropogenic causes are held responsible for mangrove degradation. However, since the last few decades, afforestation and plantation activities have been carried out in various degraded patches involving multiple stakeholders. The study has suggested 10 tier policy measures for the successful restoration of mangroves in the SBR:

1. The first steps of a restoration programme should be directed toward the identification of the environmental stressors and potential threats to restoration sites^27,108,109^. In Sundarban many past replantation programmes have failed due to a lack of proper knowledge, research communication, and documentation on mangrove ecology^110–112^. Thus, the present study recommended a holistic mangrove restoration plan by bridging ecology, society, and economics. So, setting appropriate restoration goals and creating separate standard operating procedures are essential for the restoration of different nature of degraded mangrove areas.
2. Nature-Based Solutions (NbS)^113^ for mangrove regeneration and regional seas programmes (RSP) should be indicated for innovation on mangrove regeneration^114–116^. Mangrove conservation and regeneration can be one of the best opportunities to improve the blue carbon stock which can be restored by the investment of resources for both training and building of technical and institutional capacity. NbS is a very smarter and cheap solution for restoration and conservation programmes^117^. Thus, NbS can be recommended for the Sundarban mangrove restoration programme and this can help to control stormwater intrusion and control the soil salinity in the region, which can help to improve the regeneration of mangrove species occurrence in the degraded areas.
3. Mangrove ecological integrity, community awareness, sustainable harvesting, community participation, stakeholder integration, and investment from the corporate sectors are the most important areas for the promotion of mangrove restoration and conservation^118,119^. Further, this can help to contribute to the sustainable development goals (SDGs) including inclusive and fair quality education towards mangrove restoration^120^, responsibility to make and use of mangrove resources, conservation and sustainable use of the coastal resources, protecting, restoring, and promoting sustainable ecosystems and sustainable management of forest, implementing international mangrove regeneration programme and introducing the local and global partnership to protect and restore the mangrove ecosystems.
4. The surrounding of aquaculture ponds, wetlands, and swamp areas can be considered the most useful areas for mangrove restoration. This kind of mangrove restoration approach is a great success in the coastal areas of China^121^. Some experts also advised that the restoration of coastal wetlands mangrove adopted aquaculture ponds can be helpful for future mangrove restoration^122,123^. Recently Indian Sundarbans has been declared as a Ramsar site, so restoration of coastal wetlands could be a cost-effective approach for both mangrove and wetland restoration.
5. Effective disaster and socioeconomic management schemes and strategies, and proper monitoring of mangroves can be helpful in maintaining the mangrove cover and conserving large tracts^124^. Identifying appropriate restoration techniques will be incisive for the rejuvenation of mangroves in already degraded areas. Fishbone techniques in high tidal areas and Parallel restoration techniques in low and middle tidal areas may help in restoring mangroves within the restoration prioritized areas in the Reserve^125^.
6. The habitat of Xylocarpus *mekongensis* and *Xylocarpus granatum* is restricted to a few patches within the core forest areas as these species have been targeted for illegal felling by the wood due to high price in the market^126^. Furthermost of this species has been infected by heart root disease^127^ Thus, restoration is recommended for this species within the core forest canopy gap areas.
7. Tourist willingness to pay for restoration (WTP) is an adaptive and useful approach for sustainable ecosystem restoration in Sundarbans which can help revenue generation, community engagement, livelihood options, wellbeing, and decent tourist activity for coastal ecosystems^128,129^.
8. Participatory Rural Appraisal (PRA) and Joint Forest Management (JFM) can be adopted along with the community-based mangrove reforestation (CBMR) programme and NGO initiated plantation and management within buffer and transition zones of the reserve^130–133^. Thus, the present study proposed to adopt CBMR approach along with JFM and PRA methods within the restoration prioritized areas located in the transitional zone and *Aegialitis rotundifolia, Aegiceras corniculatum, Avicennia alba, Avicennia* marina species can be adopted for this types of the restoration programme.
9. Few experimental studies have established that native grasses offered osmotic protection from tidal surges and create a favorable environment for the root establishment of mangrove species^134^:^135,136^. Thus, halotolerant grasses along with the mangrove species within the high eroded land areas and mudflat areas should be planted for successful restoration. The southern islands of the Sundarban are under serious threat due to storm surges, high tide, high wave height, erosion, and frequent cyclone. Thus, native grasses plantations can be very effective for these islands. These have been paid very less attention to mangrove regeneration compared to the northern part of the SBR.
10. Finally, the adoption of the multidisciplinary approach involving state governments, universities, research institutions, NGOs, and local organizations may provide the adaptive pathways to successful mangrove restoration in the SBR.

## Conclusion

Identification, mapping, and evaluation of the suitable habitat for mangrove species and finding restoration prioritized areas may prove an important scientific framework for sustainable mangrove conservation and protection. Our study has prepared extensive field-based species occurrence data and used 36 variables from six different parameters to develop higher accuracy habitat suitability maps for 18 true mangrove species in the Sundarbans Biosphere Reserve. We constructed ensemble models using 10 machine learning algorithms our finding identified restoration prioritized areas in the Sundarban. The findings revealed that slope and temperature have greatly influenced the mangrove habitat of the selected species. Further, human disturbance variables have also influenced the species distribution and niche lap for the Sundarban mangrove. A total of 1203 km^2^ restoration prioritize areas have been identified and prepared a species-based strategy for future restoration in the Sundarban. Strong technical and species-based knowledge is important for successful mangrove restoration^45,137^. Thus, the findings of our study will help restoration organizations in selecting species and suitable locations for the successful restoration programme. The national, state and local government authorities of India have launched different programmes for the Sundarban Biosphere Reserve for protecting, restoring and managing of the mangrove ecosystem. The Ministry of Environment, Forests and climate change of India has established a National Mangrove Committee in 1979 to protect and restore the mangrove ecosystems in India and established a large number of mangrove restoration program but most of the programme are unable to achieve their target. Thus, the present study can be helpful for understanding the nature of species distribution and a systematic way for the selection of species within the restoration prioritized areas. The findings of the present study are also in line with the ecosystem design approaches^138^, land suitability approaches^46^, and evidence-based restoration policies^48^ for scientific mangrove restoration programmes. We argue that the findings of this study can be useful for preparing a decision-making plan to prioritize conservation and mangrove restoration targets in Sundarban. The methodology adopted in this study can be applied in other geographical regions for conservation, restoration, and sustainable management of the mangrove ecosystem.

## Supplementary Information

Supplementary Information 1.

## Data Availability

All datafile produced by this study will be published in the University of Manchester official website datafile portal after publication of the main manuscript: https://www.research.manchester.ac.uk/portal/en/researchers/mehebub-sahana(e6116fb8-31a7-4c83-a3ab-fbbfea3f36bb)/datasets.html. The datasets used and/or analysed during the current study available from the corresponding author on reasonable request.
