# Supplementary material for: Assessment of suitable habitat of mangrove species for prioritizing restoration in coastal ecosystem of Sundarban Biosphere Reserve, India
Source: Sci Rep. 2022 Dec 5;12:20997. doi: 10.1038/s41598-022-24953-5 (PMC9723184; doi:10.1038/s41598-022-24953-5)
Supplement: Supplementary file 1 — Supplementary Information 1. [file 41598_2022_24953_MOESM1_ESM.docx]

**Appendix 1.** Detailed information on the parameters and the variables used for the present study

| **Parameters** | **No.** | **Variables** | **Category** | **Resolution** | **Data source** |
| --- | --- | --- | --- | --- | --- |
| **Topographical Parameters** | 1 | Elevation | class data | 30 meters | SRTM DEM, (USGS) |
|  | 2 | Slope | Continuous | 30 meters | SRTM DEM, (USGS) |
|  | 3 | Curvature | class data | 30 meters | SRTM DEM, (USGS) |
|  | 4 | Geomorphology | class data | 30 meters | LISS IV and field verification |
|  | 5 | TWI | Continuous | 30 meters | SRTM DEM, (USGS) |
|  | 6 | SPI | Continuous | 30 meters | SRTM DEM, (USGS) |
| **Environmental Parameters** | 7 | NDVI | Continuous | 30 meters | LANDSAT 8 (2020) |
|  | 8 | Level of flood inundation | class data | 30 meters | NDMA data of West Bengal |
|  | 9 | Mean tidal range | Continuous | 30 meters | Daily Tide gauge data NOAA |
|  | 10 | Storm surge height | Continuous | 30 meters | IMD India |
|  | 11 | NDMI | Continuous | 30 meters | LANDSAT 8 (2020) |
|  | 12 | Rate of Erosion/accretion | class data | 30 meters | LANDSAT MSS, TM, 8 |
| **Water Parameters** | 13 | water surface temperature | class data | 30 meters | University of Calcutta |
|  | 14 | water salinity | class data | 30 meters | Primary field survey |
|  | 15 | PH value | class data | 30 meters | Primary field survey |
|  | 16 | Distance from water | Continuous | 30 meters | Primary field survey |
|  | 17 | drainage density | Continuous | 30 meters | SRTM DEM |
|  | 18 | MNDWI | Continuous | 30 meters | LANDSAT 8 (2020) |
| **Soil Parameters** | 19 | soil texture | class data | 30 meters | NBSSLUP, India |
|  | 20 | soil salinity Index | class data | 30 meters | NBSSLUP, India |
|  | 21 | soil fertility index | Continuous | 30 meters | LANDSAT 8 (2020) |
|  | 22 | Sediment yield factor (Ton) | Continuous | 30 meters | Primary field survey for soil |
|  | 23 | Electronic conductivity (EC) | Continuous | 30 meters | Primary field survey for soil |
|  | 24 | Vegetation Soil Salinity Index (VSSI) | Continuous | 30 meters | Primary field survey for soil |
| **Bioclimatic Parameters** | 25 | BIO1 = Annual Mean Temperature | Continuous | 1000 meters | WorldClim data |
|  | 26 | BIO2 = Mean Diurnal Range | Continuous | 1000 meters | WorldClim data |
|  | 27 | BIO3 = Isothermality | Continuous | 1000 meters | WorldClim data |
|  | 28 | BIO4 = Temperature Seasonality | Continuous | 1000 meters | WorldClim data |
|  | 29 | BIO12 = Annual Precipitation | Continuous | 1000 meters | WorldClim data |
|  | 30 | BIO15 = Precipitation Seasonality | Continuous | 1000 meters | WorldClim data |
| **Disturbance Parameters** | 31 | Distance from road | Continuous | 30 meters | Open street map (OSM) |
|  | 32 | Road density | Continuous | 31 meters | Open street map (OSM) |
|  | 33 | Distance from Settlement | Continuous | 32 meters | Google earth |
|  | 34 | Population density | class data | 33 meters | census of India 2011 |
|  | 35 | Distance from agriculture field | Continuous | 34 meters | LULC map (2020) |
|  | 36 | Embankment density | class data | 35 meters | NDMA data and Google earth |


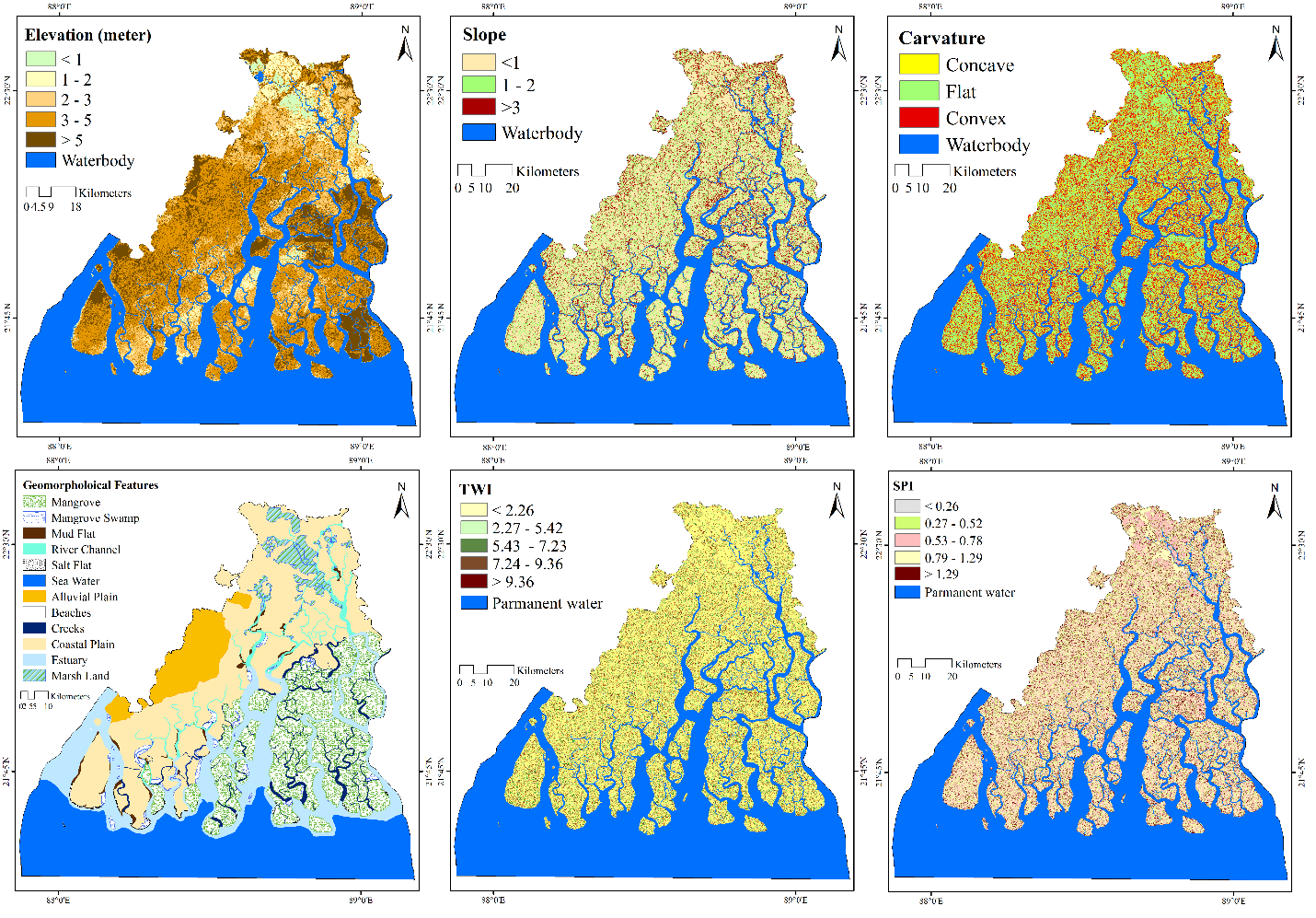


**Appendix 2A.** Selected variables under topographical parameters for habitat suitability models (The satellite images are obtained from the EarthExplorer [<https://earthexplorer.usgs.gov/>]. The maps were prepared using ERDAS IMAGINE 2014 [<https://hexagon.com/products/erdas-imagine>] and ArcGIS 10.8.2 software [<https://desktop.arcgis.com>].)


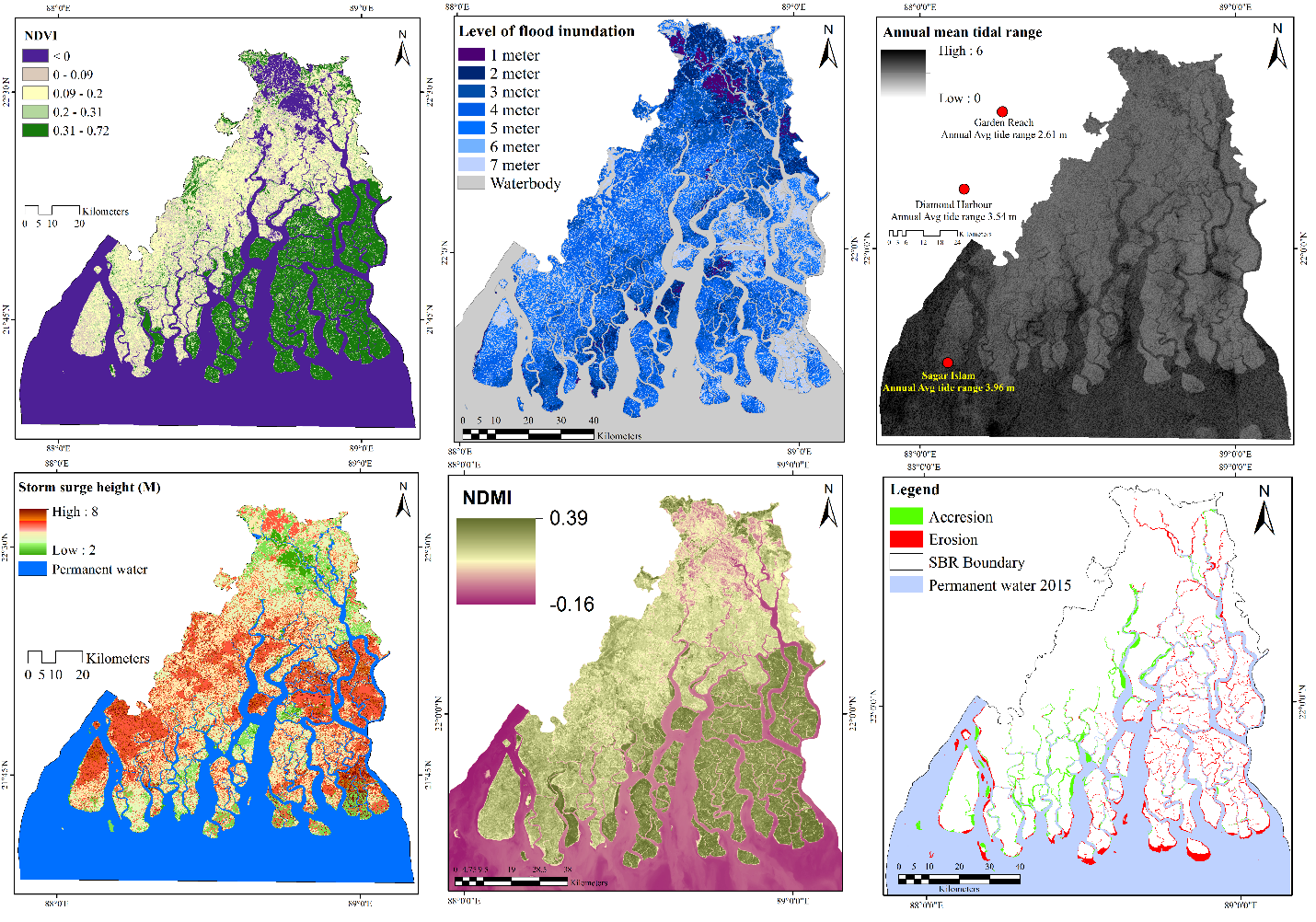


**Appendix 2B.** Selected variables under environmental parameter for habitat suitability models (The satellite images are obtained from the EarthExplorer [<https://earthexplorer.usgs.gov/>]. The maps were prepared using ERDAS IMAGINE 2014 [<https://hexagon.com/products/erdas-imagine>] and ArcGIS 10.8.2 software [<https://desktop.arcgis.com>].)


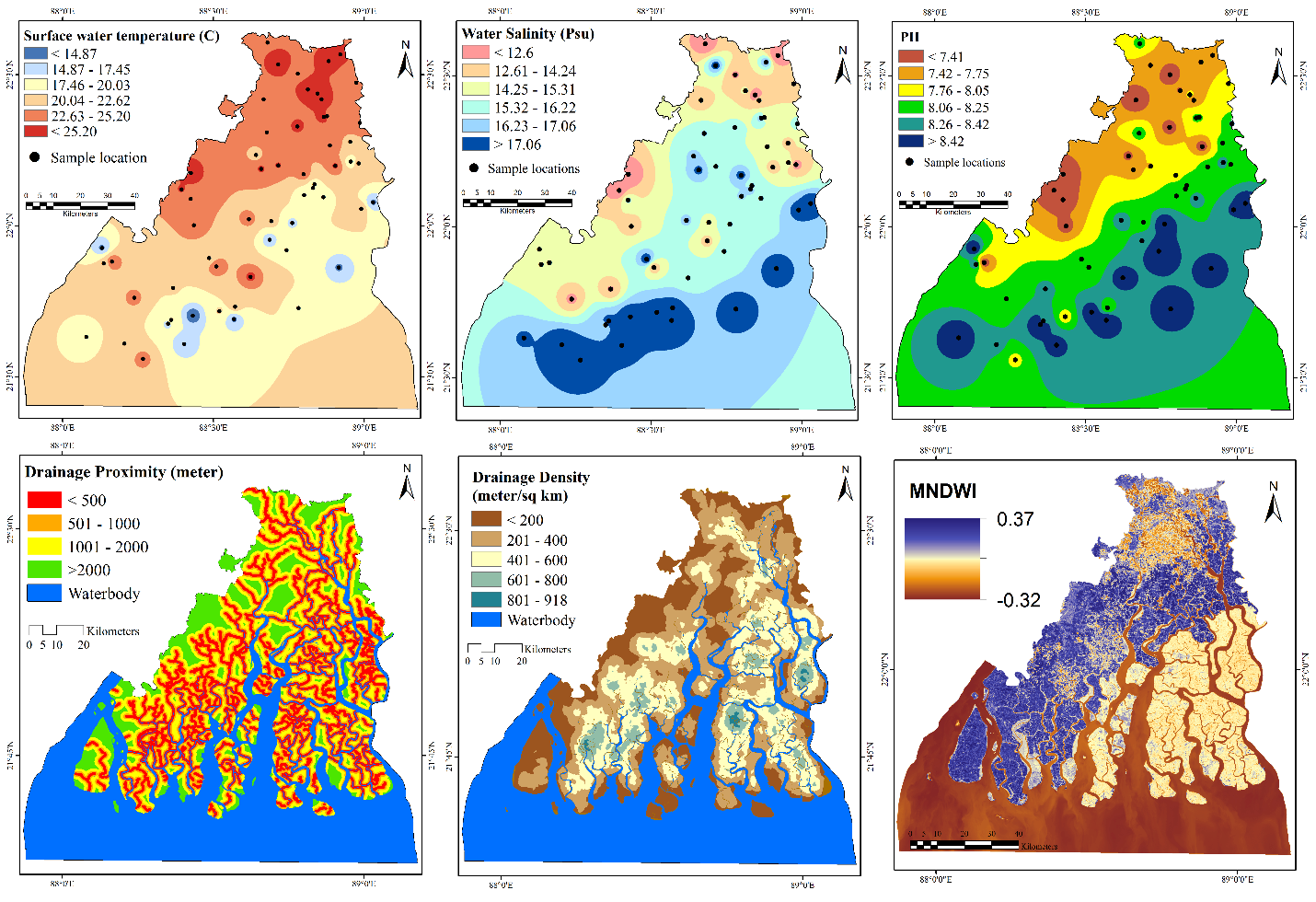


**Appendix 2C.** Selected variables under water parameter for habitat suitability models (The satellite images are obtained from the EarthExplorer [<https://earthexplorer.usgs.gov/>]. The maps were prepared using ERDAS IMAGINE 2014 [<https://hexagon.com/products/erdas-imagine>] and ArcGIS 10.8.2 software [<https://desktop.arcgis.com>].)


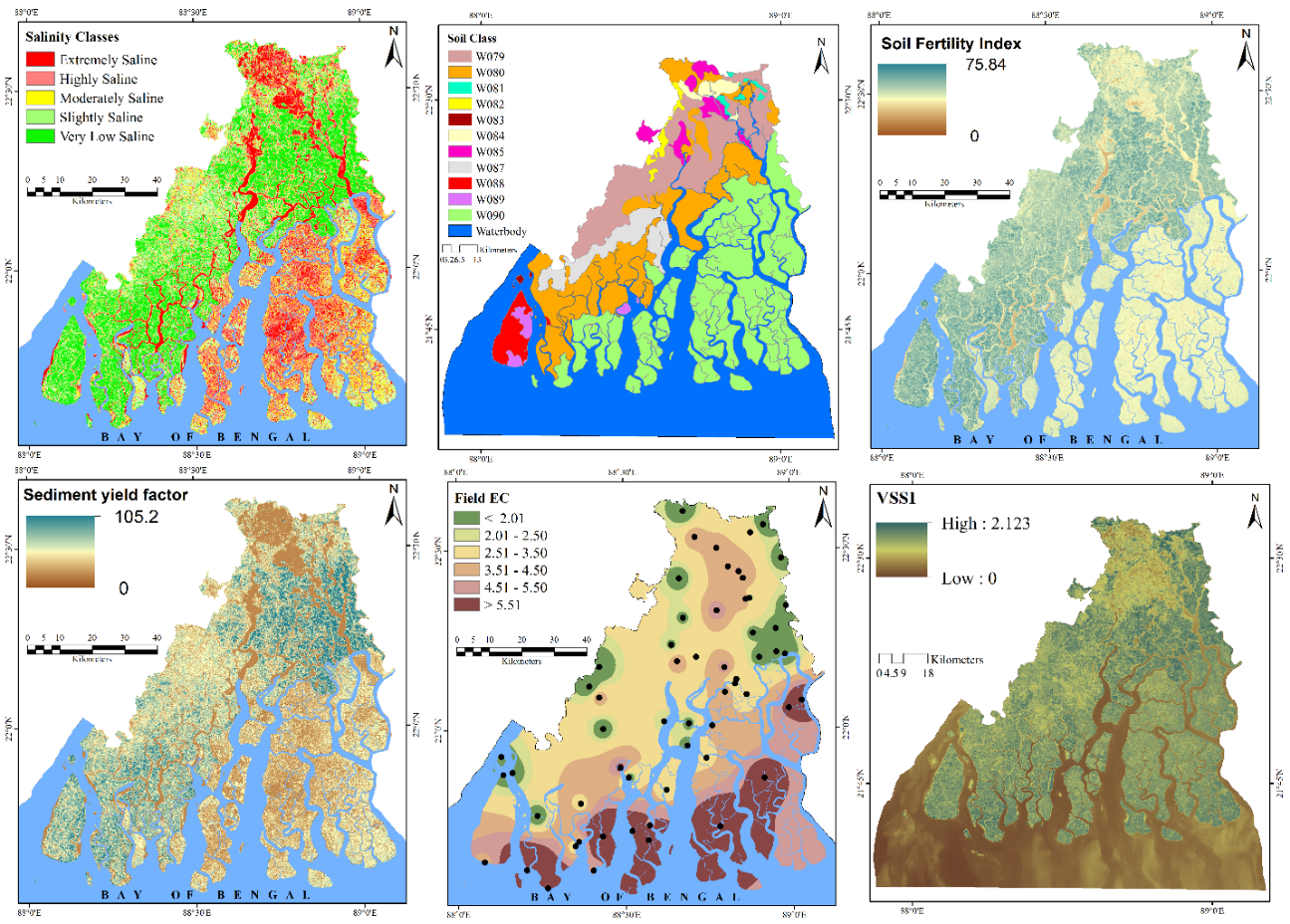


**Appendix 2D.** Selected variables under soil parameter for habitat suitability models (The satellite images are obtained from the EarthExplorer [<https://earthexplorer.usgs.gov/>]. The maps were prepared using ERDAS IMAGINE 2014 [<https://hexagon.com/products/erdas-imagine>] and ArcGIS 10.8.2 software [<https://desktop.arcgis.com>].)


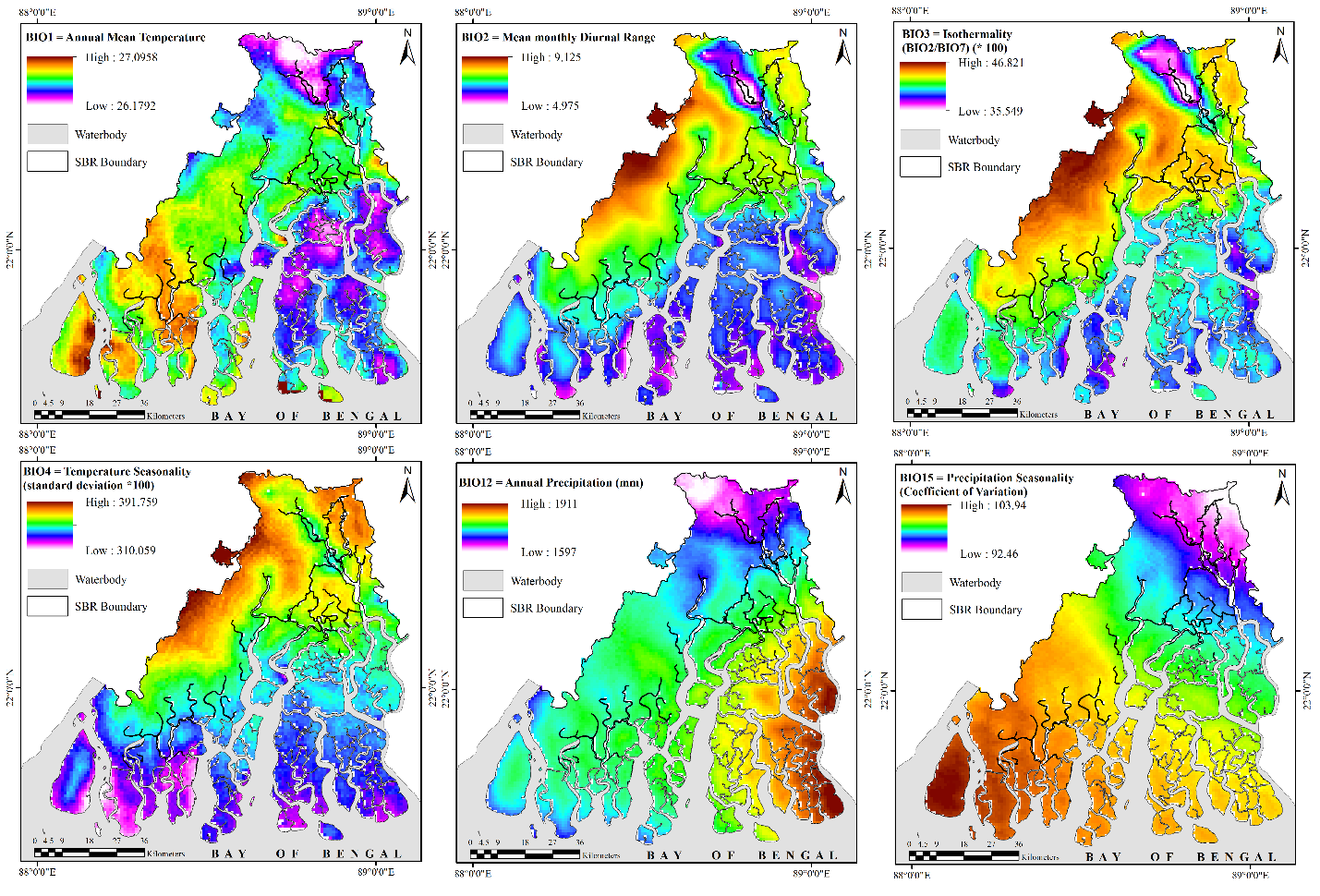


**Appendix 2E.** Selected variables under Bioclimatic parameters for habitat suitability models (The satellite images are obtained from the EarthExplorer [<https://earthexplorer.usgs.gov/>]. The maps were prepared using ERDAS IMAGINE 2014 [<https://hexagon.com/products/erdas-imagine>] and ArcGIS 10.8.2 software [<https://desktop.arcgis.com>].)


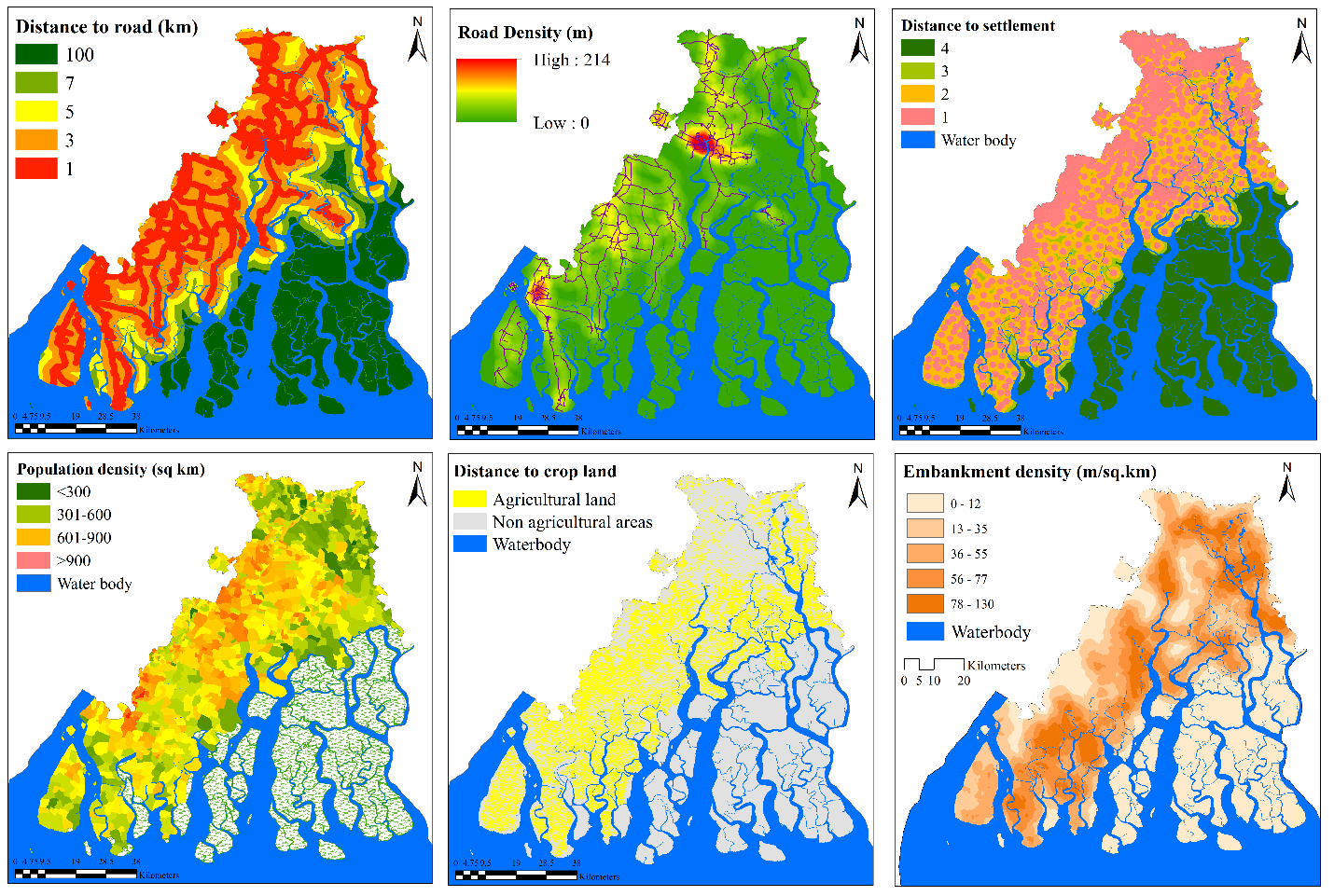


**Appendix 2F.** Selected variables under disturbance parameter for habitat suitability model (The satellite images are obtained from the EarthExplorer [<https://earthexplorer.usgs.gov/>]. The maps were prepared using ERDAS IMAGINE 2014 [<https://hexagon.com/products/erdas-imagine>] and ArcGIS 10.8.2 software [<https://desktop.arcgis.com>].)

**Appendix 3**

| Correlation matrix of bioclimatic variables | | | | | | | | | | | | | | | | | | | | |
| --- | --- | --- | --- | --- | --- | --- | --- | --- | --- | --- | --- | --- | --- | --- | --- | --- | --- | --- | --- | --- |
|  | BIO1 | BIO2 | BIO3 | BIO4 | BIO5 | BIO6 | BIO7 | BIO8 | BIO9 | BIO10 | BIO11 | BIO12 | BIO13 | BIO14 | BIO15 | BIO16 | BIO17 | BIO18 | BIO19 |  |
| BIO1 | 1 | -0.23 | 0.24 | -0.54 | 0.97 | 0.93 | -0.44 | 0.98 | 0.92 | 0.98 | 0.97 | 0.45 | 0.38 | 0.35 | -0.12 | 0.41 | 0.04 | 0.41 | 0.08 |  |
| BIO2 | -0.45 | 1 | 0.47 | 0.67 | -0.19 | -0.54 | 0.87 | -0.16 | -0.53 | -0.17 | -0.33 | -0.41 | -0.12 | -0.84 | 0.76 | -0.12 | -0.85 | -0.13 | -0.85 |  |
| BIO3 | -0.21 | 0.59 | 1 | -0.32 | 0.17 | 0.09 | 0.13 | 0.23 | 0.04 | 0.23 | 0.24 | -0.07 | 0.16 | -0.58 | 0.55 | 0.14 | -0.77 | 0.22 | -0.75 |  |
| BIO4 | -0.44 | 0.76 | -0.08 | 1 | -0.43 | -0.72 | 0.85 | -0.47 | -0.65 | -0.42 | -0.63 | -0.43 | -0.29 | -0.32 | 0.35 | -0.34 | -0.24 | -0.31 | -0.24 |  |
| BIO5 | 0.98 | -0.36 | -0.24 | -0.34 | 1 | 0.89 | -0.22 | 0.87 | 0.93 | 0.94 | 0.89 | 0.35 | 0.28 | 0.32 | -0.13 | 0.35 | 0.07 | 0.31 | 0.12 |  |
| BIO6 | 0.96 | -0.68 | -0.27 | -0.64 | 0.93 | 1 | -0.68 | 0.91 | 0.98 | 0.92 | 0.95 | 0.53 | 0.32 | 0.54 | -0.36 | 0.36 | 0.32 | 0.35 | 0.37 |  |
| BIO7 | -0.46 | 0.95 | 0.26 | 0.92 | -0.35 | -0.68 | 1 | -0.34 | -0.65 | -0.35 | -0.54 | -0.5 | -0.22 | -0.66 | 0.63 | -0.28 | -0.65 | -0.25 | -0.63 |  |
| BIO8 | 0.98 | -0.33 | -0.21 | -0.32 | 1 | 0.92 | -0.31 | 1 | 0.91 | 1 | 0.98 | 0.45 |  | 0.35 | -0.03 | 0.43 | -0.02 | 0.44 | 0.01 |  |
| BIO9 | 0.95 | -0.64 | -0.25 | -0.64 | 0.92 | 0.92 | -0.64 | 0.93 | 1 | 0.91 | 0.95 | 0.42 | 0.24 | 0.51 | -0.42 | 0.28 | 0.33 | 0.26 | 0.37 |  |
| BIO10 | 0.98 | -0.36 | -0.24 | -0.33 | 1 | 0.93 | -0.35 | 1 | 0.91 | 1 | 0.96 | 0.42 | 0.37 | 0.35 | -0.06 | 0.41 | 0.03 | 0.42 | 0.06 |  |
| BIO11 | 0.98 | -0.52 | -0.19 | -0.54 | 0.97 | 0.97 | -0.53 | 0.96 | 0.97 | 0.95 | 1 | 0.49 | 0.38 | 0.37 | -0.14 | 0.44 | 0.08 | 0.44 | 0.13 |  |
| BIO12 | 0.42 | -0.68 | -0.86 | -0.16 | 0.42 | 0.53 | -0.47 | 0.42 | 0.47 | 0.46 | 0.42 | 1 | 0.93 | 0.76 | 0.13 | 0.94 | 0.32 | 0.94 | 0.33 |  |
| BIO13 | 0.35 | -0.47 | -0.87 | 0.08 | 0.34 | 0.34 | -0.18 | 0.38 | 0.32 | 0.38 | 0.32 | 0.96 | 1 | 0.52 | 0.43 | 1 | 0.02 | 1 | -0.02 |  |
| BIO14 | 0.57 | -0.82 | -0.86 | -0.31 | 0.56 | 0.66 | -0.58 | 0.53 | 0.63 | 0.55 | 0.56 | 0.93 | 0.85 | 1 | -0.44 | 0.52 | 0.78 | 0.47 | 0.79 |  |
| BIO15 | -0.36 | 0.81 | 0.13 | 0.86 | -0.28 | -0.58 | 0.91 | -0.25 | -0.57 | -0.24 | -0.45 | -0.21 | 0.12 | -0.38 | 1 | 0.42 | -0.78 | 0.45 | -0.81 |  |
| BIO16 | 0.38 | -0.52 | -0.87 | 0.04 | 0.37 | 0.41 | -0.23 | 0.38 | 0.34 | 0.43 | 0.36 | 0.97 | 1 | 0.87 | 0.06 | 1 | 0.02 | 1 | 0.02 |  |
| BIO17 | 0.41 | -0.97 | -0.74 | -0.57 | 0.38 | 0.62 | -0.83 | 0.33 | 0.56 | 0.33 | 0.46 | 0.83 | 0.66 | 0.89 | -0.65 | 0.67 | 1 | -0.03 | 0.98 |  |
| BIO18 | 0.36 | -0.46 | -0.85 | 0.08 | 0.36 | 0.36 | -0.19 | 0.38 | 0.33 | 0.39 | 0.34 | 0.94 | 1 | 0.84 | 0.12 | 1 | 0.65 | 1 | -0.07 |  |
| BIO19 | 0.43 | -0.92 | -0.77 | -0.53 | 0.38 | 0.63 | -0.78 | 0.37 | 0.57 | 0.37 | 0.48 | 0.87 | 0.67 | 0.91 | -0.67 | 0.71 | 0.98 | 0.64 | 1 |  |

**Appendix 4.** Mean values (standard error) of AUC and TSS tests for all ten models for selected mangroves species

| **Model** | **Validation** | Species | | | | | | | | | | | | | | | | | |
| --- | --- | --- | --- | --- | --- | --- | --- | --- | --- | --- | --- | --- | --- | --- | --- | --- | --- | --- | --- |
|  |  | **AR** | **AC** | **AA** | **AM** | **AO** | **BG** | **CD** | **CT** | **EA** | **HF** | **KC** | **LR** | **NF** | **PP** | **RM** | **SA** | **XG** | **XM** |
| **GLM** | ROC | 0.866 | 0.752 | 0.903 | 0.879 | 0.882 | 0.903 | 0.752 | 0.981 | 0.732 | 0.908 | 0.619 | 0.919 | 0.841 | 0.712 | 0.967 | 0.851 | 0.751 | 0.702 |
|  | TSS | 0.862 | 0.715 | 0.754 | 0.741 | 0.896 | 0.852 | 0.841 | 0.832 | 0.741 | 0.843 | 0.751 | 0.741 | 0.841 | 0.964 | 0.865 | 0.825 | 0.810 | 0.832 |
|  | KAPPA | 0.825 | 0.841 | 0.832 | 0.736 | 0.915 | 0.985 | 0.940 | 0.847 | 0.832 | 0.741 | 0.714 | 0.951 | 0.852 | 0.841 | 0.835 | 0.789 | 0.741 | 0.765 |
| **GAM** | ROC | 0.852 | 0.847 | 0.862 | 0.821 | 0.741 | 0.856 | 0.654 | 0.870 | 0.856 | 0.874 | 0.963 | 0.714 | 0.624 | 0.952 | 0.851 | 0.954 | 0.800 | 0.954 |
|  | TSS | 0.954 | 0.984 | 0.925 | 0.957 | 0.987 | 0.782 | 0.854 | 0.641 | 0.954 | 0.952 | 0.974 | 0.821 | 0.932 | 0.841 | 0.852 | 0.658 | 0.695 | 0.852 |
|  | KAPPA | 0.685 | 0.852 | 0.721 | 0.854 | 0.951 | 0.874 | 0.741 | 0.832 | 0.824 | 0.954 | 0.714 | 0.932 | 0.987 | 0.826 | 0.742 | 0.795 | 0.815 | 0.855 |
| **GBM** | ROC | 0.910 | 0.868 | 0.914 | 0.918 | 0.935 | 0.722 | 0.803 | 0.811 | 0.843 | 0.930 | 0.797 | 0.870 | 0.809 | 0.723 | 0.910 | 0.686 | 0.914 | 0.745 |
|  | TSS | 0.846 | 0.801 | 0.686 | 0.766 | 0.851 | 0.856 | 0.622 | 0.800 | 0.810 | 0.723 | 0.809 | 0.802 | 0.772 | 0.849 | 0.850 | 0.856 | 0.764 | 0.850 |
|  | KAPPA | 0.820 | 0.918 | 0.737 | 0.906 | 0.969 | 0.828 | 0.787 | 0.737 | 0.889 | 0.953 | 0.945 | 0.877 | 0.960 | 0.635 | 0.817 | 0.727 | 0.714 | 0.854 |
| **CTA** | ROC | 0.867 | 0.820 | 0.883 | 0.731 | 0.911 | 0.910 | 0.832 | 0.880 | 0.802 | 0.860 | 0.710 | 0.913 | 0.782 | 0.731 | 0.904 | 0.871 | 0.935 | 0.748 |
|  | TSS | 0.737 | 0.777 | 0.744 | 0.823 | 0.870 | 0.753 | 0.930 | 0.891 | 0.857 | 0.816 | 0.714 | 0.861 | 0.802 | 0.891 | 0.802 | 0.785 | 0.758 | 0.776 |
|  | KAPPA | 0.876 | 0.845 | 0.886 | 0.861 | 0.722 | 0.828 | 0.573 | 0.804 | 0.834 | 0.888 | 0.823 | 0.832 | 0.738 | 0.743 | 0.888 | 0.790 | 0.734 | 0.826 |
| **ANN** | ROC | 0.867 | 0.814 | 0.731 | 0.843 | 0.833 | 0.830 | 0.865 | 0.858 | 0.802 | 0.854 | 0.749 | 0.869 | 0.750 | 0.811 | 0.900 | 0.768 | 0.855 | 0.784 |
|  | TSS | 0.871 | 0.830 | 0.862 | 0.852 | 0.777 | 0.829 | 0.818 | 0.831 | 0.818 | 0.871 | 0.786 | 0.851 | 0.744 | 0.954 | 0.894 | 0.779 | 0.833 | 0.805 |
|  | KAPPA | 0.733 | 0.885 | 0.843 | 0.880 | 0.960 | 0.851 | 0.743 | 0.785 | 0.857 | 0.954 | 0.930 | 0.905 | 0.974 | 0.731 | 0.780 | 0.761 | 0.723 | 0.855 |
| **SRE** | ROC | 0.865 | 0.893 | 0.734 | 0.912 | 0.952 | 0.835 | 0.800 | 0.847 | 0.722 | 0.942 | 0.871 | 0.873 | 0.884 | 0.706 | 0.863 | 0.706 | 0.857 | 0.815 |
|  | TSS | 0.909 | 0.939 | 0.909 | 0.934 | 0.969 | 0.809 | 0.627 | 0.707 | 0.910 | 0.947 | 0.922 | 0.847 | 0.908 | 0.818 | 0.858 | 0.818 | 0.960 | 0.834 |
|  | KAPPA | 0.882 | 0.887 | 0.888 | 0.658 | 0.900 | 0.824 | 0.782 | 0.771 | 0.865 | 0.920 | 0.860 | 0.857 | 0.845 | 0.816 | 0.872 | 0.722 | 0.771 | 0.818 |
| **FDA** | ROC | 0.862 | 0.798 | 0.814 | 0.833 | 0.888 | 0.832 | 0.722 | 0.885 | 0.787 | 0.838 | 0.712 | 0.887 | 0.725 | 0.722 | 0.906 | 0.759 | 0.899 | 0.762 |
|  | TSS | 0.798 | 0.860 | 0.635 | 0.886 | 0.943 | 0.858 | 0.772 | 0.822 | 0.834 | 0.627 | 0.856 | 0.901 | 0.898 | 0.801 | 0.846 | 0.740 | 0.728 | 0.816 |
|  | KAPPA | 0.887 | 0.951 | 0.898 | 0.931 | 0.625 | 0.805 | 0.826 | 0.689 | 0.922 | 0.953 | 0.959 | 0.849 | 0.946 | 0.738 | 0.835 | 0.865 | 0.931 | 0.853 |
| **MARS** | ROC | 0.848 | 0.882 | 0.878 | 0.883 | 0.846 | 0.828 | 0.685 | 0.770 | 0.861 | 0.920 | 0.884 | 0.854 | 0.849 | 0.689 | 0.853 | 0.883 | 0.724 | 0.840 |
|  | TSS | 0.798 | 0.860 | 0.734 | 0.886 | 0.943 | 0.858 | 0.876 | 0.822 | 0.834 | 0.942 | 0.856 | 0.901 | 0.898 | 0.801 | 0.846 | 0.740 | 0.816 | 0.654 |
|  | KAPPA | 0.746 | 0.749 | 0.863 | 0.869 | 0.860 | 0.844 | 0.907 | 0.826 | 0.826 | 0.907 | 0.821 | 0.876 | 0.821 | 0.769 | 0.870 | 0.759 | 0.876 | 0.810 |
| **RF** | ROC | 0.846 | 0.831 | 0.858 | 0.790 | 0.913 | 0.948 | 0.886 | 0.863 | 0.817 | 0.800 | 0.712 | 0.932 | 0.817 | 0.786 | 0.869 | 0.759 | 0.740 | 0.757 |
|  | TSS | 0.877 | 0.858 | 0.728 | 0.876 | 0.839 | 0.827 | 0.818 | 0.801 | 0.841 | 0.895 | 0.823 | 0.854 | 0.795 | 0.737 | 0.883 | 0.750 | 0.801 | 0.811 |
|  | KAPPA | 0.871 | 0.830 | 0.862 | 0.852 | 0.722 | 0.829 | 0.825 | 0.831 | 0.818 | 0.871 | 0.786 | 0.851 | 0.744 | 0.871 | 0.894 | 0.779 | 0.739 | 0.805 |
| **MAXENT** | ROC | 0.865 | 0.877 | 0.863 | 0.883 | 0.904 | 0.822 | 0.757 | 0.781 | 0.856 | 0.904 | 0.852 | 0.863 | 0.850 | 0.716 | 0.864 | 0.736 | 0.825 | 0.818 |
|  | TSS | 0.831 | 0.868 | 0.773 | 0.884 | 0.923 | 0.840 | 0.652 | 0.801 | 0.625 | 0.923 | 0.854 | 0.882 | 0.874 | 0.923 | 0.855 | 0.863 | 0.726 | 0.817 |
|  | KAPPA | 0.887 | 0.916 | 0.900 | 0.923 | 0.961 | 0.822 | 0.814 | 0.741 | 0.888 | 0.944 | 0.896 | 0.860 | 0.896 | 0.740 | 0.860 | 0.694 | 0.790 | 0.824 |

Note: > 0.750 represent accepted model (green colour) <0.750 represent reject models (red colour)

**Appendix 5.** Nature of mangrove degradation in Sundarban during 1975 – 2020

| **Degradation of mangrove** | **Change in sq km** | **Change in %** |
| --- | --- | --- |
| Mangrove to sand bars | 1.7 | 0.5 |
| Mangrove to Wetlands | 6.5 | 1.9 |
| Mangrove to Settlements | 8.6 | 2.5 |
| Mangrove to Swamp | 176.2 | 51.7 |
| Mangrove to Water body | 120.9 | 35.4 |
| Mangrove to Croplands | 27.0 | 7.9 |
| Total degradation | 341.0 |  |

**Appendix 6:** Species wise potential distribution of suitable habitat in SBR

| **No** | **Scientific Name** | **Local Name** | **Family** | **Inter tidal position** | **Highly suitable** | **Moderately Suitable** | **Slightly Suitable** | **Not suitable** |
| --- | --- | --- | --- | --- | --- | --- | --- | --- |
| 1 | Aegialitis rotundifolia | Tora | Plumbaginaceae | Low | 45.6 | 9.8 | 21.1 | 23.5 |
| 2 | Aegiceras corniculatum | Khalsi | Myrsinaceae | Middle | 46.9 | 8.5 | 21.9 | 22.7 |
| 3 | Avicennia alba | Kalo Baine | Acanthaceae | Low | 45.3 | 8.8 | 23.7 | 22.2 |
| 4 | Avicennia marina | Peara Baine | Acanthaceae | Middle | 44.6 | 10.3 | 24.3 | 20.8 |
| 5 | Avicennia officinalis | Jat Baine | Acanthaceae | Middle | 44.2 | 11.5 | 22.3 | 22.0 |
| 6 | Bruguiera gymnorrhiza | Bakul Kankra | Rhizophoraceae | Low | 44.7 | 8.9 | 25.7 | 20.6 |
| 7 | Ceriops decandra | Jhamti Garan | Rhizophoraceae | Low | 43.7 | 7.8 | 24.3 | 24.1 |
| 8 | Ceriops tagal | Jat Garan | Rhizophoraceae | Low | 42.1 | 10.1 | 25.0 | 22.9 |
| 9 | Excoecaria agallocha | Genwa | Euphorbiaceae | Middle, high | 30.7 | 14.9 | 27.5 | 26.9 |
| 10 | Heritiera fomes | Sundari | Malvaceae | Middle, high | 20.0 | 20.9 | 30.1 | 29.0 |
| 11 | Kandelia candel | Goria | Rhizophoraceae | Middle, high | 41.6 | 8.7 | 25.7 | 23.9 |
| 12 | Lumnitzera racemosa | Kripa/ Kripal | Combretaceae | Middle | 38.7 | 16.7 | 19.7 | 24.9 |
| 13 | Nypa fruticans | Gol Pata | Arecaceae | High | 21.8 | 19.0 | 26.0 | 33.3 |
| 14 | Phoenix paludosa | Hental | Arecaceae | Middle, High | 21.1 | 17.5 | 28.1 | 33.3 |
| 15 | Rhizophora mucronate | Garjan/ Bhara | Rhizophoraceae | Middle | 50.4 | 6.9 | 24.0 | 18.8 |
| 16 | Sonneratia apetala | Tak Keora | Lythraceae | Middle | 46.6 | 13.5 | 22.5 | 17.5 |
| 17 | Xylocarpus granatum | Dhundul | Meliaceae | Middle, High | 37.5 | 16.0 | 19.4 | 27.1 |
| 18 | Xylocarpus mekongensis | Pashur | Meliaceae | Middle, High | 32.9 | 17.7 | 21.5 | 27.9 |


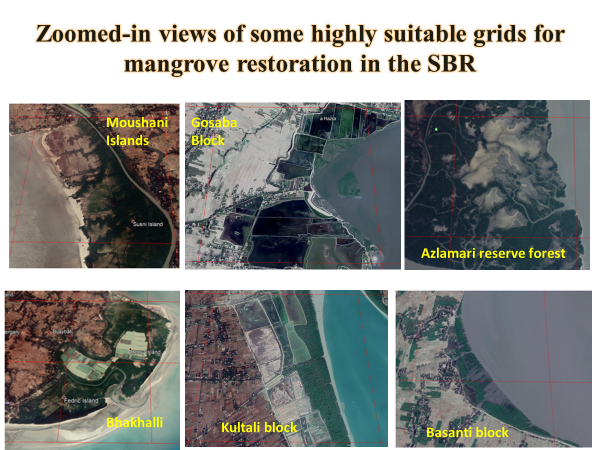


**Appendix 7:** Some specific important restoration prioritized sites as per the GRID analysis (The satellite images are obtained from the google earth pro software; version 7.3 [<https://www.google.com/intl/en_uk/earth/about/versions/>]. The maps were prepared using ArcGIS 10.8.2 software [<https://desktop.arcgis.com>].)
